# Supplementary material for: Evaluation of virtual tour in an online museum: Exhibition of Architecture of the Forbidden City
Source: PLoS One. 2022 Jan 6;17(1):e0261607. doi: 10.1371/journal.pone.0261607 (PMC8735558; doi:10.1371/journal.pone.0261607)
Supplement: S2 File — (DOCX) [file pone.0261607.s002.docx]

**Tips: the original version of the questionnaire is on p. 1-2 and the translated version is on p. 3-4.**

**北京故宫博物馆《紫禁城建筑艺术展》虚拟漫游用后评估**

谢谢您花时间和我们一起做这个调查，再开始前请仔细阅读以下说明。
**1.**在您的帮助下,我们想要了解用户对博物馆虚拟漫游的体验。
**2.**我们希望找到它的不足，这将使我们以一种尽可能高效和易于理解的方式来优化它。
**3.**体验对象：北京故宫博物馆《紫禁城建筑艺术展》虚拟漫游。
**4.** 展览主要内容是清朝宫廷建筑设计世家样式雷的成就为主题，展示故宫博物建筑类文物，包括图纸、档案、实物和模型等。
**5.**展览的形式为线上虚拟漫游，需要您在虚拟的空间中进行游览，如果您曾经对类似裸眼3D、VR、全景影像、虚拟漫游等虚拟三维空间体验有任何不适，可以放弃。
**6.**您所填写的数据仅用于本研究，并且您不需要留下您的真实姓名，本研究对个人信息完全保密。

**如果您了解以上说明，并同意参与，您可以点击：**[**《紫禁城建筑艺术展》**](https://r.wjx.com/redirect.aspx?url=https%3A%2F%2Fggzlquanjing.dpm.org.cn%2Fscene%2FgPTvX3m1LENXdkTv5UzNsDxkLU1rUNKV%2Fzijinchengjianzhuzhan%2F%2Ftour.html&activity=55096396)**，开始体验。
并请您在展览中，尝试找到展品《慈宁宫花园临溪亭秋天花》：**
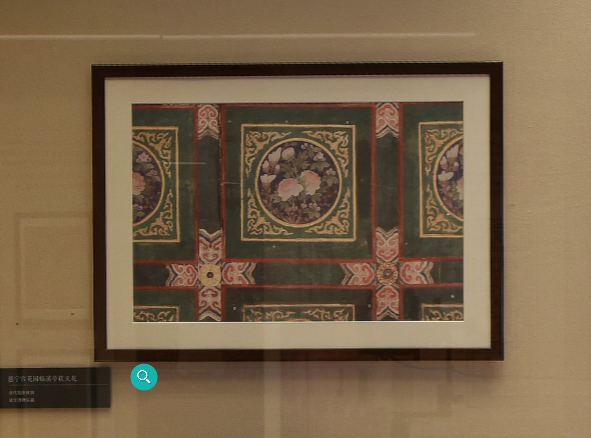
 **在您完成体验之后，即可开始填写问卷。**

1. **我感觉自己好像进入了一个真实的博物馆空间**

非常不同意□ 比较不同意□ 一般□ 比较同意□ 非常同意□

1. **展品给我的感觉很真实**

非常不同意□ 比较不同意□ 一般□ 比较同意□ 非常同意□

1. **我漫游的时候，感觉自己是在真的博物馆中餐馆**

非常不同意□ 比较不同意□ 一般□ 比较同意□ 非常同意□

1. **虚拟展览中的空间和物品对我的漫游行为的反馈很真实**

非常不同意□ 比较不同意□ 一般□ 比较同意□ 非常同意□

1. **我在虚拟漫游过程中非常自然，没有受到任何限制**

非常不同意□ 比较不同意□ 一般□ 比较同意□ 非常同意□

1. **当我移动视角时，画面变化非常自然**

非常不同意□ 比较不同意□ 一般□ 比较同意□ 非常同意□

1. **当我缩小或者放大视角时，画面变化非常自然**

非常不同意□ 比较不同意□ 一般□ 比较同意□ 非常同意□

1. **当我与展品互动时，它的反馈符合预期**

非常不同意□ 比较不同意□ 一般□ 比较同意□ 非常同意□

1. **我明白什么时候可以操作，什么时候不可以**

非常不同意□ 比较不同意□ 一般□ 比较同意□ 非常同意□

1. **我的视角变化符合我的预期**

非常不同意□ 比较不同意□ 一般□ 比较同意□ 非常同意□

1. **我始终知道自己在什么位置**

非常不同意□ 比较不同意□ 一般□ 比较同意□ 非常同意□

1. **当我迷路时，我知道如何找到自己的位置**

非常不同意□ 比较不同意□ 一般□ 比较同意□ 非常同意□

1. **我知道起点和终点的位置**

非常不同意□ 比较不同意□ 一般□ 比较同意□ 非常同意□

1. **我始终知道参观的方向**

非常不同意□ 比较不同意□ 一般□ 比较同意□ 非常同意□

1. **我能够得到我想得到的信息**

非常不同意□ 比较不同意□ 一般□ 比较同意□ 非常同意□

1. **我在展览中获取足够多的信息**

非常不同意□ 比较不同意□ 一般□ 比较同意□ 非常同意□

1. **我得到的信息让我觉得有趣**

非常不同意□ 比较不同意□ 一般□ 比较同意□ 非常同意□

1. **我会和别人讨论我获得的信息**

非常不同意□ 比较不同意□ 一般□ 比较同意□ 非常同意□

**Evaluation of Virtual Roaming in an Online Museum:**

**Exhibition of Architecture of the Forbidden City**

**Thank you for taking the time to do this survey with us, please read the following instructions carefully before you begin.**

1. With your help, we would to understand users' experience of virtual roaming of the online museum.
2. We want to find its weaknesses, which will allow us to optimize it in a way that is as efficient and easy to understand as possible.
3. Object: Exhibition of Architecture of the Forbidden City.
4. The content of the exhibition is the achievements of Lei, an royal architectural designer of the Qing Dynasty, and it displays the architectural relics of the Palace Museum, including drawings, archives, objects and models.
5. The exhibition is in the form of online virtual roaming, which requires you to travel in a virtual space. If you have any discomfort with the virtual 3d space experience such as naked eye 3D, VR, panoramic image, virtual roaming, etc., you can give up.
6. The data you have filled in will only be used for this study, and you are not required to leave your real name. Personal information will be kept strictly confidential in this study.

**If you understand the above instructions and agree to participate, you can click on "**[**Exhibition of Architecture of the Forbidden City**](https://ggzlquanjing.dpm.org.cn/scene/gPTvX3m1LENXdkTv5UzNsDxkLU1rUNKV/zijinchengjianzhuzhan/tour.html)**" to start.**

**
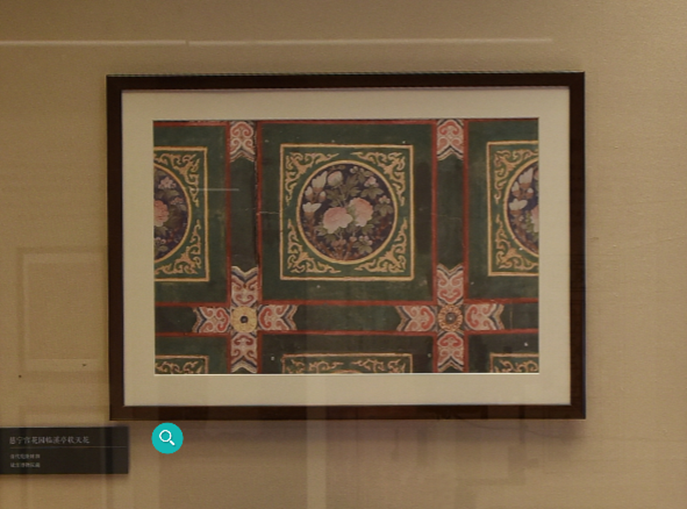
**

**You can begin to fill out the questionnaire After completing the experience.**

1. **When I wander, I feel like I’m in a real museum.**

Strongly disagree□ Disagree□ General□ Agree□ Strongly agree□

1. **I felt like I was in a real museum**

Strongly disagree□ Disagree□ General□ Agree□ Strongly agree□

1. **The artifacts give me a very real feeling**

Strongly disagree□ Disagree□ General□ Agree□ Strongly agree□

1. **The space and objects in the virtual exhibit give real responses to my wandering behavior**

Strongly disagree□ Disagree□ General□ Agree□ Strongly agree□

1. **The process of my virtual roaming is very natural and there are no restrictions**

Strongly disagree□ Disagree□ General□ Agree□ Strongly agree□

1. **When I move the camera, the picture changes very naturally**

Strongly disagree□ Disagree□ General□ Agree□ Strongly agree□

1. **When I zoom out or zoom in, the picture changes very naturally**

Strongly disagree□ Disagree□ General□ Agree□ Strongly agree□

1. **I understand what I can operate and what I can’t**

Strongly disagree□ Disagree□ General□ Agree□ Strongly agree□

1. **When I interacted with the exhibits, the feedback was as expected**

Strongly disagree□ Disagree□ General□ Agree□ Strongly agree□

1. **My perspective changed in line with my expectations**

Strongly disagree□ Disagree□ General□ Agree□ Strongly agree□

1. **I always knew the directions to visit**

Strongly disagree□ Disagree□ General□ Agree□ Strongly agree□

1. **I always know where I am**

Strongly disagree□ Disagree□ General□ Agree□ Strongly agree□

1. **I know how to locate myself when I am lost**

Strongly disagree□ Disagree□ General□ Agree□ Strongly agree□

1. **I know where we start and where we end**

Strongly disagree□ Disagree□ General□ Agree□ Strongly agree□

1. **I can get the information I want**

Strongly disagree□ Disagree□ General□ Agree□ Strongly agree□

1. **I get enough information from the exhibition**

Strongly disagree□ Disagree□ General□ Agree□ Strongly agree□

1. **The information I get is interesting to me**

Strongly disagree□ Disagree□ General□ Agree□ Strongly agree□

1. **I will discuss the information with others**

Strongly disagree□ Disagree□ General□ Agree□ Strongly agree□
