# Supplementary material for: Evaluation of virtual tour in an online museum: Exhibition of Architecture of the Forbidden City
Source: PLoS One. 2022 Jan 6;17(1):e0261607. doi: 10.1371/journal.pone.0261607 (PMC8735558; doi:10.1371/journal.pone.0261607)
Supplement: S3 File — (DOCX) [file pone.0261607.s003.docx]

| NO. C1 | Gender | Age | | | | | Time  2020.3.5 |
| --- | --- | --- | --- | --- | --- | --- | --- |
|  | Female√ Male | ≤19 | 20-29 | 30-39√ | 40-49 | ≥50 |  |
| **1.The overall feeling**   1. How did you feel about the experience?   感觉还可以，只能满足基本的体验，没有惊喜。  The feeling is OK, can only satisfy the basic experience, no surprise.  Further question: why no surprise？  因为它展示的在博物馆中也可以看到，没什么惊喜，或超出意料的。换句话说，它可以被看作是是以一个博物馆展览，但是没有超越博物馆的传统感受。但是好处也有，就是你可以在网上反复观看展品而不需要到现场。  Because it shows what you can see in a museum, nothing surprising, nothing out of the ordinary. In other words, it can be seen as a museum exhibition, but without transcending the traditional feel of a museum. However, it also has advantages, that is, you can watch the artifacts repeatedly without having to go to there.   1. What were some of the problems you encountered?   不太好用，不方便，尤其是上楼时，半天都没有成功上楼。  Inconvenient, especially when upstairs, half a day did not succeed upstairs.  Further question: How did you get upstairs later?  试了很多遍才成功。  I tried many times before I made it. | | | | | | | |
| **2. Authenticity**   1. A：Do you feel real?   还不错，我觉得满分10分的话可以打7-8分  Not bad. I'd say 7-8 out of 10.   1. Does it feel like visiting a real museum exhibition?   像，我觉得可以打8分，如果满分是10的话。  yes, It feels real to me. I think it's an 8 out of 10.  Further question: What is the reason for the deduction?  移动的时候不能随心所欲  You can't move as you want totally. | | | | | | | |
| **3. Interactivity**   1. How do you feel about interacting with the virtual exhibition?   还不错，基本上能满足我看展的需求。从一个点到另一个点转换连贯性不足，楼下和楼上的连续性不足，可能是导览不够简明吧。  Not bad. It basically meets my exhibition needs. Lack of continuity from one point to another, lack of continuity from downstairs to upstairs, perhaps because the tour is not concise enough.  Further question：Have you tried the function buttons at the bottom? Does it work?  用过，一般。要是有具象化的导览就更好了。  Yes, just so-so, an embodying guide would be better.   1. How do you feel the virtual exhibition respond to you?   回馈不错，能了解一些展览的信息的，但缺点是展览方式传统，看久了会有倦怠感。  The feedback is good, I can understand some information about the exhibition, but the disadvantage is that the exhibition is traditional, so I will feel tired after watching it for a long time. | | | | | | | |
| **4. Navigation**   1. Do you have any trouble finding directions or exits?   有困难，尤其是方向和上楼的方面，找出口还好。  Have difficulty, especially with directions and going upstairs, finding the exit is fine.   1. Do you know where you are in the exhibition?   知道的，我就觉得自己上楼比较困难。  Yes I know. I just found it hard to get upstairs by myself. | | | | | | | |
| **5. Learning**   1. Do you think you can learn anything from this exhibition?   我学到了，了解这些展品的名称、基本见解，我觉得我会到现场去看。但是，介绍的方式比较难找，比较累，在信息方面都是以文字为主，看久了会疲倦。  I learned that, knowing the names of the exhibits, the basic ideas, I felt I would go and see them on site. However, the introduction is difficult to find, more tired, in terms of information are based on text, look for a long time will be tired.   1. Did you find the exhibition interesting?   还不错，总体符合展览主题，也能了解展览的基本信息，，音乐也不错，我觉得不错。  Not bad, generally in line with the theme of the exhibition, but also to understand the basic information of the exhibition, background music is not bad, I think it is good. | | | | | | | |

| NO. B1 | Gender | Age ：29 | | | | | Time：3.5 |
| --- | --- | --- | --- | --- | --- | --- | --- |
|  | Female Male√ | ≤19 | 20-29√ | 30-39 | 40-49 | ≥50 |  |
| **1.The overall feeling**   1. How did you feel about the experience?   有一定现实应用意义，可以为很多偏远地区提供美学教育，但是还是得做好，不然体验感不好，意义就会降低。  It has a certain practical application significance and can provide aesthetic education for many remote areas. However, it should be done well, otherwise the experience will be bad and the significance will be reduced.  Further question: 具体说下呢？  还不错，就是有些地方不太会走，还有就是最好能把3d效果做到更好些。  Not bad, but some places are not very good, and it is better to do a better 3D effect.   1. What were some of the problems you encountered?   还好，不过我没有体验过其他的，是我的第一次体验，主要问题可能就是3D的感觉一般。OK, but I haven't experienced anything else, it's my first experience, and the main problem is probably that the 3D feels just so-so. | | | | | | | |
| **2. Authenticity**   1. A：Do you feel real?   真实度方面还是可以的，我有在里面的感觉，但是这种感觉是隐约的，就像我之前说的，3D效果可以再加强一些。  The realism was fine, I had the feeling of being in it, but it was a bit vague, and as I said before, the 3D effect could be enhanced a bit more.   1. Does it feel like visiting a real museum exhibition?   某些画面细节还是很到位的，它有一定真实的感觉。  Some picture details are very good, it has a certain authenticity to it. | | | | | | | |
| **3. Interactivity**   1. How do you feel about interacting with the virtual exhibition?   互动还算细致，但是可以增加一些互动，比如加入一些动画。  The interaction is high-resolution, but you can add some interaction, such as animations.  Further question：high-resolution?  对，就是放大看起来很清晰。  Yes, the zoom in to make it look good.   1. How do you feel the virtual exhibition respond to you?   还可以，这些互动已经足够了，对我来说是够了，我对这些本来不够了解，打开信息看完后有一定了解。  Not bad, these interactions are enough, it is enough for me, I did not understand these originally, opened the information after reading certain understanding. | | | | | | | |
| **4. Navigation**   1. Do you have any trouble finding directions or exits?   有困难，可能我的方向感不强，但是我感觉很困难，我没法随心所欲参观。  Yes, maybe I don't have a good sense of direction, but I feel very difficult. I can't go around as I want.   1. Do you know where you are in the exhibition?   我大概知道自己在几楼，但是在什么位置不是很清晰，还好博物馆不是很大，要是非常大，我更要考虑这个问题，不然找不到自己的位置。  I probably know which floor I am on, but where I am is not very clear. Fortunately, the museum is not very big. If it is very big, I will consider this problem more, otherwise I can't find my position | | | | | | | |
| **5. Learning**   1. Do you think you can learn anything from this exhibition?   肯定学到了一些  I must have learned something  Further question: Can you tell me more about that?  参观完我对这个展示技术很感触，我之前没有看过类似的。  After the visit, I was very impressed with the demonstration technology, I had never seen anything like it before.  Further question: Did you learn anything about the artifacts?  在看展的过程中，我也学到了展品的知识。  In the process of visiting the exhibition, I also learned the knowledge of the artifacts.   1. Did you find the exhibition interesting?   我觉得还需要增加趣味性，比如增加视频或者其他一些有特色的东西，最好可以跟不同受众结合，比如孩子喜欢的卡通的元素，在快乐中学习。  I think it is necessary to add more fun, such as adding videos or other characteristic things. It is better to combine with different audiences, such as cartoon elements that children like, so that they can learn in happiness. | | | | | | | |

| NO.  C2 | Gender | Age | | | | | Time：3.5 |
| --- | --- | --- | --- | --- | --- | --- | --- |
|  | Female√ Male | ≤19 | 20-29 | 30-39√ | 40-49 | ≥50 |  |
| **1.The overall feeling**   1. How did you feel about the experience?   很好啊，我觉得很有意思啊，我之前上网看车时候也玩过这个，没想到博物馆还可以这样。  That's nice. I think it's very interesting. I've played this before when I was looking at cars online.   1. What were some of the problems you encountered?   问题吗？还好，没什么太大问题，可能就是展览太小了，虽然看起来很丰富，但是展品信息太少，看不到太多东西。  Fortunately, there is nothing serious. Maybe the exhibition is just too small. Although it looks rich, there is too little information on the exhibits to see much. | | | | | | | |
| **2. Authenticity**   1. A：Do you feel real?   不错啊，感觉挺清晰挺真实的，放大也足够清楚的，比我之前用的更清晰。  It's nice. It feels nice and real, and it zooms in clearly enough. It's sharper than what I used before.   1. Does it feel like visiting a real museum exhibition?   展品还行，里面有些文字的，还有花纹，我看不太清，要是能放更大些就好了。  The exhibits are all right. There are some words and patterns in them, but I can't read them clearly. I wish they were bigger. | | | | | | | |
| **3. Interactivity**   1. How do you feel about interacting with the virtual exhibition?   互动还行，嗯，一般吧。  Further question：具体说下呢？  点开来信息也太少了，哪怕多放点放大的图也好。  There is too little information to click on, even if there are more magnified images.   1. How do you feel the virtual exhibition respond to you?   对我的反应吗？感觉有时候太灵敏，画面有时候转得太快。其他都还好，还是信息太少，只有文字，（互动）可以再多一点。之前,我在看汽车的虚拟漫游时，我可以换颜色  Sometimes the feeling is too sensitive, sometimes the screen turns too fast, other things are ok, or too little information, only text, interaction can be a little more. Before, when I visited the virtual roaming of the car, I could change cars’ colors. | | | | | | | |
| **4. Navigation**   1. Do you have any trouble finding directions or exits?   嗯，有时候我会不太清楚我已经参观过哪些（展品）了，开始时候我会重复看展品，我不喜欢这一点。  Yes, sometimes I'm not quite sure what I've seen, and at the beginning I looked at the artifacts repeatedly, which I don't like.  Further question：为什么会重复呢Why is it repeated？  可能是我不太会用，但是那个箭头我偶尔会搞混淆，他们看起来一样，我不喜欢这个设计，至少我想知道哪个是下一个场景。  Maybe I'm not very good with it, but I get confused with that arrow occasionally, they look the same, I don't like this design, at least I want to know which scene is next..   1. Do you know where you are in the exhibition?   知道的，大致的在哪里我还知道的，后来你会发现这个展览不大，还是简单的  Yes, where do I know. Later you will find that the exhibition is not big, and simple. | | | | | | | |
| **5. Learning**   1. Do you think you can learn anything from this exhibition?   有学到的  Further question具体说下吗？  你能看到一些信息，而且我觉得我以后可以在这个里面先看看展览有没有感兴趣的东西，再去现场看看。  You can see some information, and I think I can look in this to see if there is anything interesting in the exhibition, and then go to the site.   1. Did you find the exhibition interesting?   还是很有意思的，我是觉得在家里看到北京的博物馆展览就很有趣。  It's very interesting. I think it's interesting to see the museum exhibition at home.  Further question：展品信息有意思吗？  我对这个主题还是有点兴趣，但是我觉得信息太简单了，而且描述的太枯燥，我不喜欢这点。  I'm still somewhat interested in the subject, but I think the information is too simple and the description is too dry, which I don't like it. | | | | | | | |

| NO.D1 | Gender | Age | | | | | Time：3.5 |
| --- | --- | --- | --- | --- | --- | --- | --- |
|  | Female√ Male | ≤19 | 20-29 | 30-39 | 40-49√ | ≥50 |  |
| **1.The overall feeling**   1. How did you feel about the experience?   我觉得很好的。  I think it's good.   1. What were some of the problems you encountered?   我觉得好像视觉切换有点奇怪吧，  I think the visual switch is a little weird.  Further question：能具体说说吗？Can you tell me more about it?  我说不太清楚，就是一开始看起来感觉很真实，但是参观时候有点不自然。  I can't put my finger on it, but it looked and felt real at first, but it was a little unnatural during the tour. | | | | | | | |
| **2. Authenticity**   1. A：Do you feel real?   看起来真实，这很好。  It looks real. It's good.  Further question：Does participation feel real?  还不错，大部分看起来很真实，图片质量也不错。  Not bad, mostly it looks real and the quality of the pictures is good.   1. Does it feel like visiting a real museum exhibition?   像的，感觉很真实，那个画面感觉应该是现场拍摄的吧，有点像百度地图导航的一项功能。yes, the feeling is very real, that picture feeling should be shot on the spot, a bit like a function of Baidu Map navigation. | | | | | | | |
| **3. Interactivity**   1. How do you feel about interacting with the virtual exhibition?   还好，用起来不难。  Not bad, it's not hard to use.   1. How do you feel the virtual exhibition respond to you?   感觉不太自然，每当我看下一个场景的时候不太自然，好像不是我想看的东西，我是觉得场景切换不太自然的，就像我之前说的我感觉有点奇怪。  It doesn't feel natural, every time I look at the next scene it doesn't feel natural, it doesn't seem like something I want to see, I feel like the scene switches don't feel natura，it's a little weird as I said before. | | | | | | | |
| **4. Navigation**   1. Do you have any trouble finding directions or exits?   还行。  not too bad  Further question：能具体说说吗Can you tell me more about it?  因为好像就是一个回字形的空间而已，再加上两层楼，比较简单，但是方向上有点乱，我在二楼绕了两次才顺利下楼。  Because it seems to be a “回” shape of the space, and two floors, relatively simple, but the direction of a little messy, I was on the second floor around twice before smooth downstairs.   1. Do you know where you are in the exhibition?   知道的，因为空间比较简单。  Yes, because the space is easy. | | | | | | | |
| **5. Learning**   1. Do you think you can learn anything from this exhibition?   学到了一些与传统建筑有关的知识，尤其是样式雷，我第一次知道原来中国古代还有个皇家设计师，这是我第一次听说。  I learned some knowledge about traditional architecture, especially the style Lei and I learned for the first time that there was a royal designer in ancient China.   1. Did you find the exhibition interesting?   有意思，画面很清晰，色彩也很丰富，我还喜欢那个背景音乐。  It's interesting. The pictures are clear and the colors are rich. I also like the background music. | | | | | | | |

| NO.C3 | Gender ：female | Age ：30-39 | | | | | Time：3.6 |
| --- | --- | --- | --- | --- | --- | --- | --- |
|  | Female√ Male | ≤19 | 20-29 | 30-39√ | 40-49 | ≥50 |  |
| **1.The overall feeling**   1. How did you feel about the experience?   还不错的，我喜欢去博物馆。  It's not bad. I like going to museums.   1. What were some of the problems you encountered?   我觉得好像没遇到什么大问题。  I don't feel like I'm having any big problems. | | | | | | | |
| **2. Authenticity**   1. A：Do you feel real?   挺真实的啊，感觉真的有在现场啊。我觉得这个环境挺好的，我下次想亲自去看看。  It's real. It feels like I'm there. I think the environment is very nice, and I want to go there myself next time.   1. Does it feel like visiting a real museum exhibition?   是的，挺像真的博物馆的，这是一个美丽的博物馆。  Yes, it's like a real museum. It's a beautiful museum. | | | | | | | |
| **3. Interactivity**   1. How do you feel about interacting with the virtual exhibition?   互动的话感觉一般，展品感觉就是图片和文字吧，我想看的更详细些。  The interactive words feel general, the artifacts feel only the picture and text, I want to see more details.   1. How do you feel the virtual exhibition respond to you?   简单了点，就只有文字和图片信息，还好图片够清晰，但是要是更清晰一些就好了，有的文字也不清晰，比如前言里的，还有很多展板上的文字，其实我对这些信息很感兴趣。  A little simple, just text and picture information. I'm glad the picture is clear enough, but I wish it were clearer. Some text is not clear, such as in the preface or panels, which I am very interested in.  Further question：你觉得应该有哪些互动。What kind of interactions?  可以放视频之类的，或者是细节放大的图片，这个展览是很清晰，但是我觉得还不够。  It can play videos or pictures with enlarged details. This exhibition is very clear, but I think it is not enough. | | | | | | | |
| **4. Navigation**   1. Do you have any trouble finding directions or exits?   我觉得我在这个空间方向感不好，可能因为我一直以来空间感都不好。  I don't think I have a good sense of direction in this space, probably because I've always had a bad sense of space.  Further question：Bad sense of direction?  这个空间对我来说有点变形，尤其是转向的时候，感觉到不自然，我会搞不清楚下一步看哪个，我以前玩3d游戏就不太喜欢虚拟空间，我想我更想去现场。  This space is a little distorted for me, especially when I turn it, it feels unnatural, and I don't know what to look at next. I used to play 3D games and I didn't really like virtual Spaces. I think I'd rather be on site.   1. Do you know where you are in the exhibition?   嗯，我知道我在哪里，但其实最好有实时更新的小的导航图。  Yes, I know where I am, but it's better to have a small navigation map that's updated in real time. | | | | | | | |
| **5. Learning**   1. Do you think you can learn anything from this exhibition?   是的，有学到的，我虽然不是这个专业，但是里面的信息还是很感兴趣，并且我没事就可以反复看。  Yes, I have learned something. Although I am not a major in this field, I am still very interested in the information in it, and I can read it again and again.   1. Did you find the exhibition interesting?   我觉得有意思，展品还是很丰富的。  I found it interesting. The artifacts are very rich. | | | | | | | |

| NO.D2 | Gender | Age ：40-49 | | | | | Time：3.6 |
| --- | --- | --- | --- | --- | --- | --- | --- |
|  | Female Male√ | ≤19 | 20-29 | 30-39 | 40-49√ | ≥50 |  |
| **1.The overall feeling**   1. How did you feel about the experience?   我感觉还行，但是我觉得还不够好。  I feel OK, but I don't think it's good enough.   1. What were some of the problems you encountered?   我觉得还不能自由的探索，受到展览的限制，比如好像路线很呆板，不够自由。  I don't think I can explore freely. I am restricted by the exhibition. For example, the route seems to be rigid and not free enough. | | | | | | | |
| **2. Authenticity**   1. A：Do you feel real?   这个感觉很真实的，我觉得这个应该不是虚拟的，这个是真实的场景的影像吧。  This exhibition feels real, I don't think this is virtual and is the image of the real scene.   1. Does it feel like visiting a real museum exhibition?   是的，这个比较像真实的博物馆  Yes, this is more like a real museum | | | | | | | |
| **3. Interactivity**   1. How do you feel about interacting with the virtual exhibition?   还不坏，但是我觉得互动还可以更多。那个蓝色的放大镜点开后让我很失望，有的只有标题，信息太少，而且我居然没办法把那个信息的页面关掉。  Not bad, but I think there could be more interaction. When the blue magnifying glass was opened, I was very disappointed, because some was only the title, not enough information, and I can't even close the information.   1. How do you feel the virtual exhibition respond to you?   就像我刚才说的，我觉得回应的还不够，比如图片或者更详细信息。  As I said, I think there should been more response as pictures or more detailed information. | | | | | | | |
| **4. Navigation**   1. Do you have any trouble finding directions or exits?   我在方向上有点不舒服，但是出口没问题。  I'm a little uncomfortable with the direction, but the exit is no problem.  Further question：Uncomfortable？  方向的变化跟我想的不一样，我觉得它的变化是被安排好的，我点击那个方向，但是它并没有按照我想的变化，这让我有点晕。  The direction change is not what I thought it was going to be, I think it's going to be arranged.  I click in that direction, but it doesn't go the way I want it to, and that makes me dizzy.   1. Do you know where you are in the exhibition?   知道，这个展览的结构比较简单，所以我大概知道我自己在什么位置。  Yes, the structure of the exhibition is relatively simple, so I know where I am roughly. | | | | | | | |
| **5. Learning**   1. Do you think you can learn anything from this exhibition?   我觉得我学到不多，就像我之前说的，信息太少了，而且有些描述太专业了导致我看不懂。  I don't think I've learned much. Like I said before, there's too little information, and some of the descriptions are too professional for me to understand.   1. Did you find the exhibition interesting?   还行吧，展览整体体验还是蛮有意思的，展品也挺精美，而且毕竟我可以不用到现场参观。  Not bad, the overall experience of the exhibition is interesting, the exhibits and artifacts are fineness, and after all, I don't need to go to the site to visit. | | | | | | | |

| NO. E1 | Gender | Age ： | | | | | Time：3.6 |
| --- | --- | --- | --- | --- | --- | --- | --- |
|  | Female Male√ | ≤19 | 20-29 | 30-39 | 40-49 | ≥50√ |  |
| **1.The overall feeling**   1. How did you feel about the experience?   我觉得还是可以的，但是我觉得这得有点专业知识才看得懂。  I think it's OK, but I think it takes some expertise to understand it.  Further question: 你的体验的感觉如何？  这个很是很好的，如果你看得懂的话，就会觉得会很好的。但是，没有现场看的实物好。不过，这个展览比较便捷一些，不方便去现场的人可以在网上看。  This is very good, if you can understand it, it will be very good. However, there is no spot to see the real good. However, this exhibition is a little more convenient, people who are not convenient to go to the site can see it online.   1. What were some of the problems you encountered?   看展没什么问题，就是路线上操作不太便捷，第一次尝试会原地转圈，操作上好像有问题。  There was no problem with the exhibition, but the operation on the route was not convenient. I turned in circles at the first attempt, but there seemed to be something wrong with the operation. | | | | | | | |
| **2. Authenticity**   1. A：Do you feel real?   这倒是很像真实的东西，看着很真实，有身临其境的感觉，像是在现场看的东西。  It was like something real. It looked real. It felt like something you were watching on the spot.   1. Does it feel like visiting a real museum exhibition?   这还是像真实博物馆的，就是缺少一个讲解员，如果有讲解员就更像了。  It's like a real museum, except that it lacks a docent, and it would be even more like a docent. | | | | | | | |
| **3. Interactivity**   1. How do you feel about interacting with the virtual exhibition?   这些展览都是静态的，好像没什么互动。  The exhibitions are static, and there seems to be little interaction.  Further question：上面有蓝色的放大镜符号的点，你有点开试试吗？  我有发现这些点，它弹出来的内容我看不太清楚，我戴着眼镜也看不太清楚。  I found these dots. I can't see what it's popping up, but I can't see them very clearly with my glasses.   1. How do you feel the virtual exhibition respond to you?   好像不能像我想象那样反应。比如我想往里走，它总是会回到原来的画面。这里有两个箭头，我击它，它应该给我直观地往里走才对，但是它总是重复给我一个画面。  I don't seem to react the way I thought I would. If I want to go inside, it always comes back to the original picture. I have two arrows here, and if I hit it, it should give me an intuitive way to go in, but it always gives me the same picture. | | | | | | | |
| **4. Navigation**   1. Do you have any trouble finding directions or exits?   我可能操作不太熟练，不过还是那个箭头的问题，它应该走向它指示的方向，但是它总是转向另一个方向，转向另一个画面。  I may not be very good at this, but it's the same arrow, it's supposed to go in the direction it's supposed to go, but it keeps going the other way, to another picture.   1. Do you know where you are in the exhibition?   这个我知道，正因为我知道自己在什么位置，我才知道自己没走到我想去的位置。  I know that. Because I know where I am, I know I'm not where I want to be. | | | | | | | |
| **5. Learning**   1. Do you think you can learn anything from this exhibition?   这个展览能够让人获得一些知识，能够学到一些东西。  The exhibition can be informative, and you can learn something.   1. Did you find the exhibition interesting?   我认为这个不是一般人看得懂的，不了解的人只能随便看看，不会有很强烈的印象。  I don't think this is something that ordinary people can understand. People who don't know it can only read it casually, and they won't have a strong impression. | | | | | | | |

| NO. B2 | Gender | Age | | | | | Time：3.7 |
| --- | --- | --- | --- | --- | --- | --- | --- |
|  | Female Male√ | ≤19 | 20-29√ | 30-39 | 40-49 | ≥50√ |  |
| **1.The overall feeling**   1. How did you feel about the experience?   我觉得还不错啊，我喜欢这个设计，我以前没体验过，打开的时候让我觉得很惊艳。  I think it's good, I like the design, I've never experienced it before, it's amazing when it opens.  Further question：为什么觉得很惊艳Why do you think it's amazing  因为画质很清晰，音乐也很动听，这个展览给我的感觉很好，我没想到展览可以这样看，我蛮喜欢的。  Because the picture quality is very clear and the music is very beautiful, this exhibition gives me a very good feeling, I didn't expect the exhibition to visit like this, I really like it.   1. What were some of the problems you encountered?   我好像没遇到什么大问题。  I don't seem to be having any major problems. | | | | | | | |
| **2. Authenticity**   1. A：Do you feel real?   嗯，我觉得挺真实的。  Yes, I think it's real.   1. Does it feel like visiting a real museum exhibition?   这确实像一个真的博物馆，这个画面很清晰，空间感也很强烈，我觉得我在一个真的博物馆里参观。  It looks like a real museum, and the picture is very clear, and the sense of space is very strong, I feel like I'm visiting a real museum. | | | | | | | |
| **3. Interactivity**   1. How do you feel about interacting with the virtual exhibition?   互动的感觉还好，我感觉还可以再多一些。那些2D画还好，但是立体的展品我觉得我想看得更多角度。点开的时候最好不只是文字信息，如果有一个虚拟的模型可以跟我互动，让我可以旋转或者放大它就更好了。  The interaction was good, I felt like I could use a little bit more of that. The 2d paintings were fine, but the 3d exhibits I felt I wanted to see from more angles. It's better to have more than just a text message when you click on it. It's even better to have a virtual model that I can interact with and rotate or zoom in on.   1. How do you feel the virtual exhibition respond to you?   对我的反应还不错啊，就是切换画面的时候感觉不够快。  The respond to me was good, but I didn't feel fast enough when I switched scenes.  Further question: what about the respond of the artifacts or exhibition space itself?  展览的空间还可以啊，我就是觉得展品的反应可以更多些，如果再加一个人物的形象作为导览员就好了，或者至少有语音导览。  The exhibition space is OK. I just think the exhibits could be more responsive. It would be nice to have a human figure as a guide, or at least a voice guide. | | | | | | | |
| **4. Navigation**   1. Do you have any trouble finding directions or exits?   嗯，可以，这些没有什么太大问题。  Yes, that's not too much of a problem.   1. Do you know where you are in the exhibition?   还行，要是有地图就好了。  Not bad. I wish I had a map.  Further question：There is a map function in the lower left corner. Have you noticed that?  没有，我没注意到那个功能啊  No, I didn't notice that function. | | | | | | | |
| **5. Learning**   1. Do you think you can learn anything from this exhibition?   嗯，我觉得能学到一些知识，展览已经提供了一些知识，但是我觉得还不够啊。就像我之前说的，要是有更多信息就好了，哪怕是提供展品的链接也好。  I think I can learn some knowledge, and the exhibition has provided some knowledge, but I don't think it's enough. As I said before, it would be nice to have more information, even if it was to provide links to the exhibits.   1. Did you find the exhibition interesting?   还好，我只是觉得展品的信息形式太简单了。我有仔细看过这个展览，其他都还好，展品本身都还不错的。  Not bad, I just think the information form of the artifacts is too simple. I have looked at the exhibition carefully and everything else is fine. The exhibits themselves are quite good. | | | | | | | |

| NO. B3 | Gender | Age | | | | | Time：3.7 |
| --- | --- | --- | --- | --- | --- | --- | --- |
|  | Female√ Male | ≤19 | 20-29√ | 30-39 | 40-49 | ≥50 |  |
| **1.The overall feeling**   1. How did you feel about the experience?   我喜欢这个看展览的方式，我觉得只要有网络就可以看，这是很有意思的功能。而且我觉这个功能对需要的人很有帮助，也能节省时间和金钱。  I like the way to see the exhibition, I think if there is Internet can see, this is a very interesting function. And I think it's helpful for people who need it, and it saves time and money.   1. What were some of the problems you encountered?   我不喜欢这些展品的，因为环境看起来很有立体感，但是展品感觉没有立体感，这让我觉得奇怪。  I don't like these artifacts, because the environment looks very 3D, and the artifacts feel no 3D, which makes me feel strange. | | | | | | | |
| **2. Authenticity**   1. A：Do you feel real?   我感觉环境的真实感比较强，其他的就一般了。  I feel the reality of the environment is relatively strong, the other is general.   1. Does it feel like visiting a real museum exhibition?   是的，这像在博物馆里，环境的立体感还是很强烈的，环境很干净，有音乐却没有噪音，这个让我觉得很棒。  Yes, it's like being in a museum. The 3D feeling of the environment is very strong. The environment is clean, with music but no noise, which makes me feel good. | | | | | | | |
| **3. Interactivity**   1. How do you feel about interacting with the virtual exhibition?   我觉得互动不多，展品就像一张张照片，虽然是在虚拟的环境中，但是我觉得我只不过再看照片而已。  I don't think there is much interaction. The exhibits are just like photos. Although they are in a virtual environment, I think I just look at the photos again.  Further question：Does participation feel real?  看起来真实，放大后就是照片，我没有感觉到和展品有什么真实的互动。  It just looks real. When I enlarge the visions, they are just photos, and I don't feel any real interaction with the exhibits.   1. How do you feel the virtual exhibition respond to you?   还不错，我的觉得展览的环境基本上都能理解我的行为，也能做出我想要的回应，就是那个箭头我有时候找不到它。  It's not bad. I think the environment of the exhibition basically understands my behavior and responds to it in the way that I want, but the arrow that I sometimes can't find. | | | | | | | |
| **4. Navigation**   1. Do you have any trouble finding directions or exits?   不太好，我觉得画面有点变形而且反应有点奇怪，尤其是在旋转方向的时候，我得慢慢习惯这种变化。我没有看到最后，我不知道出口在哪里。  Not so good, I think the image is a little distorted and a little strange, especially in the direction of rotation. I'll have to get used to it. I didn't see the end, and I didn't know where the exit was.  Further question：你为什么没看完？Why didn't you finish it?  我对这个主题没什么兴趣，而且展品都感觉差不多。  I'm not interested in the subject, and the exhibits all feel the same.   1. Do you know where you are in the exhibition?   我没有在意这件事，我只是顺着箭头方向前进。  I didn't pay attention to it. I just followed the direction of the arrow. | | | | | | | |
| **5. Learning**   1. Do you think you can learn anything from this exhibition?   还行，展览的信息对我来说足够多了，太多我是看不下去的。  Not bad. There's enough information about the exhibition for me. It's too much for me to read.   1. Did you find the exhibition interesting?   还行吧，我对这个主题不是很了解，我觉得还是很有趣的，因为我觉得这个线上的展示方式本身就很有趣。因为我们离北京很远，这样看展览的方式让我就不去北京就可以看这个展览，也就不用怕有很多人妨碍你，因此你也不用担心其他人会影响你在博物馆里看展，特别是游客特别多的时候。  Not bad, I don't know much about the subject, but I think it's interesting, because I think the online presentation itself is very interesting. Since we are far away from Beijing, this way of seeing the exhibition allows me to see the exhibition without going to Beijing, so that I don't have to be afraid of many people interfering with you, so you don't have to worry about other people influencing your viewing in the museum, especially when there are a lot of tourists. | | | | | | | |

| NO.B4 | Gender | Age | | | | | Time：3.7 |
| --- | --- | --- | --- | --- | --- | --- | --- |
|  | Female√ Male | ≤19 | 20-29√ | 30-39 | 40-49 | ≥50 |  |
| **1.The overall feeling**   1. How did you feel about the experience?   我觉得还行，不是很好用。  I think it's OK. It doesn't work very well.  Further question：能说下哪里不好用吗？  我觉得设计的逻辑不太好，比如我想去哪里，但是我总是被发现自己到了另外一个视角。  I don't think the logic of the design is very good, like where I want to go, but I always find myself in a different perspective.   1. What were some of the problems you encountered?   Further question：其他问题能说说吗？  其他我一开始没找到音乐关闭按钮，我觉得哪些按钮的符号有点奇怪，因此我一个个尝试试才知道功能。  I didn't find the music off button at first, I thought the symbols of which buttons were a little strange, so I tried one by one to find out the function. | | | | | | | |
| **2. Authenticity**   1. A：Do you feel real?   我觉得还是挺真实的，画面很清晰，这让我觉得真实。  I think it's very real, it's very clear, it makes me feel real.   1. Does it feel like visiting a real museum exhibition?   这个确实像一个真实的博物馆，看起来都很真实。  It does look like a real museum. It looks real. | | | | | | | |
| **3. Interactivity**   1. How do you feel about interacting with the virtual exhibition?   我就是觉得它总不能带我到我想看的角度，尤其那个箭头，好像总会把我引导一个和我之前不太一样的视角。  I just felt like it didn't get me the perspective I wanted to see, especially the arrow, which always seemed to lead me to a different perspective than I had before   1. How do you feel the virtual exhibition respond to you?   还行，对我的反应还是挺快的。  Not bad, I was pretty quick to react to.  Further question：what about the artifacts?  还行啊，我觉得要是能看到更多角度就好了，现在的展品能看的角度不好，有的文字放得视角不好，我读不到。  Not bad, I think it would be good if I could see more perspectives, the present exhibits cannot be seen from a good viewing angle, and some words are placed at a bad location, I cannot read it clearly. | | | | | | | |
| **4. Navigation**   1. Do you have any trouble finding directions or exits?   还行，多试几次我大概搞清楚它的方向的切换逻辑，我想强调的是我觉得看展品的角度太单一了，我想看到更多角度。  It's OK. I'll try it a few times, so I can figure out the switching logic of its direction. What I want to emphasize is that I think the Angle of viewing the exhibits is too single, and I want to see more perspectives.   1. Do you know where you are in the exhibition?   我基本上知道自己在哪里，我有个模糊的概念，但是我不知道精确的，得看下面的小地图。  I basically know where I am, I have a vague idea, but I don't know exactly, I must look at the mini-map below. | | | | | | | |
| **5. Learning**   1. Do you think you can learn anything from this exhibition?   我对展览的信息印象不是很深刻，感觉弹出的展板信息太少了，看展品的角度也不好，好像没什么可以学的。  I am not very impressed with the information of the exhibition. I feel that there is too little information on the pop-up panels and the perspectives of the exhibits is not good enough. It seems that there is nothing to learn.   1. Did you find the exhibition interesting?   还行吧，整体的感觉还行，这个是我第一次参观这种展览，还是蛮有意思的。  Not bad, the overall feeling is not bad, this is my first time to visit this kind of exhibition, it is quite interesting. | | | | | | | |

| NO.B5 | Gender | Age | | | | | Time：  3.7 |
| --- | --- | --- | --- | --- | --- | --- | --- |
|  | Female Male√ | ≤19 | 20-29√ | 30-39 | 40-49 | ≥50 |  |
| **1.The overall feeling**   1. How did you feel about the experience?   还不错，我觉得这种看展览的感觉挺好。  Not bad. I think it's a good feeling to see the exhibition.   1. What were some of the problems you encountered?   遇到的问题就是看展的路线我觉得有问题，好像总是让我走回头路，我不知道为什么，我觉得偶尔有时候会在原地打转。  The problem I encountered was that I felt there was something wrong with the route of the exhibition. It always seemed to make me go back. I don't know why, but I felt that sometimes I would turn in circles. | | | | | | | |
| **2. Authenticity**   1. A：Do you feel real?   真实感不错，有身临其境的感觉。  It feels real，and it feels like you're actually there.   1. Does it feel like visiting a real museum exhibition?   虽然看起来真实，但是跟真实的博物馆比我觉得还有点距离，至少我看展的路线让我觉得受到很大限制。而且好像也没有其他人一起看，这个感觉有点不真实。和这个比，我更喜欢到现场去看，因为在现场我感觉更自由。  Although it looks real, it is still a little far from the real museum, and at least the way I see the exhibition makes me feel very limited. And there seemed to be no one else to watch it with, which felt a little surreal. Compared to this, I prefer to go to the scene because I feel freer there. | | | | | | | |
| **3. Interactivity**   1. How do you feel about interacting with the virtual exhibition?   我感觉互动不多，因为展品好像没有提供什么互动。  I don't feel much interaction because the exhibits don't seem to offer much interaction.   1. How do you feel the virtual exhibition respond to you?   我觉回应还足，展览对我的回应太简单。我觉得就是不够自由，没法想到哪里就去哪里。  I think the response is not enough. The exhibition's response to me is too simple, and I just don't feel free enough to go where I want. | | | | | | | |
| **4. Navigation**   1. Do you have any trouble finding directions or exits?   有的，我觉得我在看展的时候，有两三次在原地打转。我觉得这个界面设计不够好，它应该有个地图现实我在什么位置，我至少可以不需要只能顺着它设计的路线走。  Yes, I think I walked in circles two or three times when I was looking at the exhibition. I think the interface design is not good enough, it should have a map to show where I am, I can at least not need to have to follow the route designed by it.   1. Do you know where you are in the exhibition?   一开始还好吧，总体来说我是搞不太清楚我在哪里，要是有地图就好了。  It was OK at first, but in general I didn't really know where I was. I wish I had a map.  Further question：您有发现底下有地图的功能按钮吗？Did you find the function button with the map at the bottom?  有地图吗？我没有看到它在哪里。  Does it have a map? I don't see where it is. | | | | | | | |
| **5. Learning**   1. Do you think you can learn anything from this exhibition?   我了解一些古代建筑的情况，算是学到一些吧，但是信息如果更丰富一些就好了。  I know a little bit about ancient architecture, but I wish I had more information.   1. Did you find the exhibition interesting?   还行吧，我觉得要是有更多趣味性的元素就好了，这个展览开起来太官方了。我有一个女儿，我常带她去看博物馆，但是这种博物馆她应该不会喜欢去。  Not bad. I think it would be nice if there were more interesting elements. This exhibition is too official. I have a daughter, and I often take her to museums, but this is not the kind of museum she would like to go to. | | | | | | | |

| NO. B6 | Gender | Age | | | | | Time：3.8 |
| --- | --- | --- | --- | --- | --- | --- | --- |
|  | Female√ Male | ≤19 | 20-29√ | 30-39 | 40-49 | ≥50 |  |
| **1.The overall feeling**   1. How did you feel about the experience?   很不错啊，我觉得很不错，整个空间看起来很精美。  It's very nice. I think it's very nice. The whole space looks very nice.   1. What were some of the problems you encountered?   问题吗？我觉得太小了，这个展览不够大。还有就是画面切换不够流畅，这可能是线上的展览原因吧。但是我觉得这些问题都不大，但不影响我，  I think it's too small. This exhibition isn't big enough. There is also not smooth screen switching, which may be the reason for the online exhibition. But I don't think these problems are big enough to affect me. | | | | | | | |
| **2. Authenticity**   1. A：Do you feel real?   嗯，挺真实的啊，整个氛围营造的很好、很逼真。尤其是建筑的很多细节，比如说天花板，我可以放得很大看到细节，这让我觉得很好。  Yes, it's very real, the whole atmosphere is very good and realistic. Especially a lot of the details of the building, like the ceiling, I can zoom in and see the details, which makes me feel good.   1. Does it feel like visiting a real museum exhibition?   像是在参观真的博物馆，还不错。但是有一点不好，就是我没法围着建筑模型看，我想看看那个建筑模型的侧面，但是好像没法看到，这个应该改进一些。  It's like visiting a real museum. Not bad. But one thing is bad, I can't look around the building modal, I want to see the side of them, but I can't seem to see it, this should be improved. | | | | | | | |
| **3. Interactivity**   1. How do you feel about interacting with the virtual exhibition?   互动有点不够真实，就是说我没法按照我想看的方式去看这些展品，我觉得最好提供一个可以随意旋转的模型会比较好。  The interaction was a little unreal. I meant I couldn't see the exhibits as my will, and I thought it would be better to provide a model that could be rotated at will.   1. How do you feel the virtual exhibition respond to you?   我感觉展览对我的回应比较简单吧。还有一点，我旋转的时候我感觉视觉比较模糊，这让我觉得不真实。  I feel that the exhibition is responding to me too simple. Also, my vision is blurred when I rotate, which makes me feel unreal. | | | | | | | |
| **4. Navigation**   1. Do you have any trouble finding directions or exits?   还好，我觉得不是很难找到。  Not hardly, I don't think it's too hard to find.   1. Do you know where you are in the exhibition?   差不多吧，这个展览也就两侧楼，我大概知道自己在哪里。  Yes, the exhibition is just two floors away, so I know where I am. | | | | | | | |
| **5. Learning**   1. Do you think you can learn anything from this exhibition?   我觉得还行，我不是都能看懂，不过我也会顺手上网查。  I think it's OK, I don't understand everything, but I also look it up on the Internet.   1. Did you find the exhibition interesting?   说实话我不是很懂这里的知识，虽然展品很丰富，但是都是文字我觉得看着有点累，要是有视频我可能会看得更久。但是总体来说，这个展示形式很好玩，我第一次看这种展览，我觉得还是很有趣的。  To be frank, I am not very familiar with the knowledge here. Although the exhibits are very rich, they are all text, which makes me feel a little tired. If there are videos, I may visit for longer. But overall, it's a very interesting museum, and it's the first time I've seen this kind of exhibition, and I think it's very interesting. | | | | | | | |

| NO. B7 | Gender | Age | | | | | Time：3.8 |
| --- | --- | --- | --- | --- | --- | --- | --- |
|  | Female√ Male | ≤19 | 20-29√ | 30-39 | 40-49 | ≥50 |  |
| **1.The overall feeling**   1. How did you feel about the experience?   我觉得这个很好，我喜欢这种展览，尤其这么精致的画质和音乐。  I think this one is very good. I like this kind of exhibition, especially the fine picture quality and the music.   1. What were some of the problems you encountered?   我觉得还好，没有发现什么问题。  I think it's fine. I didn't find anything wrong. | | | | | | | |
| **2. Authenticity**   1. A：Do you feel real?   是的，挺真实。  Yes.  Further question：能具体讲讲这种感觉吗？Can you tell me more about that feeling?  你会感觉你在一个很精美的博物馆里参观，你甚至可以放大，很多东西都能看得很清楚，这种真实感很强烈。  You feel like you are in a beautiful museum, you can even zoom in, you can see a lot of things clearly, this sense of reality is very strong.   1. Does it feel like visiting a real museum exhibition?   是的，我这种感觉特别好，而且你会感觉你就在这个建筑里面，我之前没有玩过VR，但是听说过，这个感觉特别不错。  Yes, it's a great feeling for me, and you feel like you're in the building, and I haven't played VR before, but I've heard of it, and it's a great feeling. | | | | | | | |
| **3. Interactivity**   1. How do you feel about interacting with the virtual exhibition?   互动的话还行，我发现展品的信息比较简单，所以我后来没有怎么点开展品信息，我觉得展品本身就很多信息。  The interaction was ok, but I found the information on the exhibits relatively simple, so I didn't click the information of exhibits afterwards.   1. How do you feel the virtual exhibition respond to you?   我觉得还不错啊，这个空间感觉很真实，但是我知道这是虚拟的，所以这个比只看图片好多了，很多网上展览都只有图片和文字，这个感觉有走在其中的感觉。  I think it's pretty good. This space feels real and I know it's virtual, so it's much better than just looking at pictures. Many online exhibitions only have pictures and words, so I like the feeling that I'm walking in the middle of it. | | | | | | | |
| **4. Navigation**   1. Do you have any trouble finding directions or exits?   还行，我没有遇到什么困难，感觉一开始有点乱，但是慢慢你就找到规律了，可能是我对这种虚拟现实不熟悉的愿意吧。  Not bad, I didn't encounter any difficulties, I felt a little confused at the beginning, but slowly you find the rules, maybe I am not familiar with this kind of virtual reality is willing  Further question：What's the rules?  你每次点击下一步时候，你的视角会被改变。  Each time you click on the next step, your perspective changes.   1. Do you know where you are in the exhibition?   我没想过这个问题，我就是顺着它的路线参观。  I hadn't thought about it. I was just following its route | | | | | | | |
| **5. Learning**   1. Do you think you can learn anything from this exhibition?   我觉得能学到一些东西，和在博物馆看起来差不多，没啥区别。  I think I can learn something. It looks the same as in a museum.   1. Did you find the exhibition interesting?   有趣的，这种虚拟现实我觉得很有意思。  Yes, this kind of VR I think is very interesting. | | | | | | | |

| NO. B8 | Gender | Age | | | | | Time：3.8 |
| --- | --- | --- | --- | --- | --- | --- | --- |
|  | Female Male√ | ≤19 | 20-29√ | 30-39 | 40-49 | ≥50 |  |
| **1.The overall feeling**   1. How did you feel about the experience?   还行，我觉得还不够好，我不知道如何表述我的感觉，我觉得这个展览让我觉得不自然。我感觉我在一个博物馆，但是行为又不像在一个现实的博物馆里。我觉得这更像一个游戏吧，就是那种根据你的行为变换场景的游戏，而不是可以自由行动的三维空间游戏。  I don't think it's good enough, I don't know how to describe my feeling, I don't think the exhibition made me feel natural I feel like I'm in a museum, but the behavior is not like in a realistic museum. I think it's more like a game, where you change the scene based on your behavior, rather than a 3d space game where you can move freely.   1. What were some of the problems you encountered?   我觉得行为不自然，我没法自由行动。  I don't feel natural. I can't move freely. | | | | | | | |
| **2. Authenticity**   1. A：Do you feel real?   我感觉这看起来还是比较真实的，这点确实不错。  I feel like it looks real, and that's a good feature.   1. Does it feel like visiting a real museum exhibition?   不像的，我觉得真实的博物馆应该可以很自由的参观展品，这个虽然看起来很真实，但是没什么自由度，甚至还不如博物馆里的展品清晰。在博物馆我可以凑近了看，并且还可以借个语音导览器，这个不行。总之和真实的博物馆差距有点大。  No, I think a real museum should be free to visit the exhibits. Although it looks real, there is little freedom, and even it is not as clear as the artifacts in the museum. At the museum I can get a closer look, and I can borrow a voice guide, but not this one. Anyway, it's a little different from the real museum. | | | | | | | |
| **3. Interactivity**   1. How do you feel about interacting with the virtual exhibition?   我觉得缺乏互动。我之前也说了，这个博物馆虽然看起来真实，但是缺少那种真正的可以和展品互动的感觉。比如我没法围绕中间的展品仔细看，我通常在博物馆我会关注展品的细节。我觉得跟这个虚拟博物馆相比，我更喜欢到现场看展览。这个只能临时看看，不能取代真实的博物馆。  I feel a lack of interaction. As I said before, the museum looks real, but it lacks the sense of real interaction with the exhibits. For example, I can't look around the exhibits in the center, and I usually pay attention to the details of the exhibits in the museum. I think I prefer to see the exhibition on site to this virtual museum. This is a temporary visit, not a substitute for a real museum.   1. How do you feel the virtual exhibition respond to you?   我觉得回应的很少，我跟展品互动太少了，就是这个原因让我觉得这个展览一般。  I think there was very little response. I didn't interact with the artifacts very much, and that's why I think the exhibition was so mediocre. | | | | | | | |
| **4. Navigation**   1. Do you have any trouble finding directions or exits?   在这个方面我好像没遇到什么麻烦，我没有特别在意方向和出口，我觉得顺着他们的箭头就好了。  I didn't seem to have any trouble with that. I didn't care much about directions and exits, and I thought I'd just follow their arrows.   1. Do you know where you are in the exhibition?   我大概知道。这个展览好像有两层，而且路线也比较简单，我能大概知道自己在什么位置。  I sort of know. The exhibition seems to have two floors, and the route is relatively simple, so I can roughly know where I am. | | | | | | | |
| **5. Learning**   1. Do you think you can learn anything from this exhibition?   我觉得可以学到一些东西，这个展览的展品还是很丰富的。  I think I can learn something. The exhibits in this exhibition are very rich.   1. Did you find the exhibition interesting?   我觉得没什么太多趣味性吧，这个展览展品很多，但是展示的方式很一般，跟普通博物馆差不多啊。我觉得其实它可以做得更有趣一些，比如增加一些视频或者游戏。这个展览本身就像个游戏，但是却缺少互动且太学术化，所以会有点枯燥。展览完全可以做成像游戏一样啊，有一个卡通人物跟我对话，那会有意思的多。  I don't think it's much interesting. There are a lot of items in the exhibition, but the way it's display is old. It's just liked an ordinary museum. I think it could have been more fun, like adding some videos or games. The exhibition itself is like a game, but the lack of interaction and too academic makes it a bit boring. The exhibition could be made into a game, with a cartoon character talking to me. That would be much more interesting. | | | | | | | |

| NO. C4 | Gender | Age | | | | | Time：3.8 |
| --- | --- | --- | --- | --- | --- | --- | --- |
|  | Female Male√ | ≤19 | 20-29 | 30-39√ | 40-49 | ≥50 |  |
| **1.The overall feeling**   1. How did you feel about the experience?   我认为还不错。这种不在现场能看到展览的感觉很特别，我觉得很奇妙。我自己很喜欢看博物馆，但是我平时比较忙，所以我觉得这是一种选择。  I think it's good and it's a very special feeling to be able to see the exhibition without being there and I think it's amazing. I love museums myself but I'm usually quite busy, so I think it's an option.   1. What were some of the problems you encountered?   我感觉没遇到什么问题。  I don't feel any problem. | | | | | | | |
| **2. Authenticity**   1. A：Do you feel real?   嗯，我感觉还行，感觉挺真实的。  Yes, it feels real.   1. Does it feel like visiting a real museum exhibition?   怎么说呢，我觉得这有点矛盾，它看起来确实很像在一个博物馆，但是又感觉不现实。你知道，在博物馆里有很多人，有噪音，有走动的感觉。在这里我感觉不到自己在走动。  Well, I think it's a bit of a contradiction, it does look like being in a museum, but it doesn't feel realistic you know, there are a lot of people in a museum, there's noise, there's a sense of moving around and I don't feel like I'm moving around here. | | | | | | | |
| **3. Interactivity**   1. How do you feel about interacting with the virtual exhibition?   我觉得还行吧，他们看起来挺真实的。展品放大看还是能看到一些细节，但是有些东西我看不清楚，比如有些字太小。  I think it's ok. They look real. When I zoom in, I can still see some details, but I can't make out some things, for example, some of the words are too small.   1. How do you feel the virtual exhibition respond to you?   感觉有回应，但是还没有现实博物馆那种回应的真实感。  There is a sense of response, but there is not yet the sense of authenticity of the response of a real museum.  Further question：回应的真实感是什么？What is the authenticity of the response?  我不太能说清楚，这只是我的感觉。比如在一个真正博物馆里，你可以感受很多外在的东西回应，比如走路的脚步声，轻声对话声，还有多媒体的声音。我觉得这里只有音乐，点开展品也只有文字太简单了点，要是有更多元素可能让我的沉浸感更强烈。  I'm not quite sure. It's just my feeling. In a real museum, for example, you can feel a lot of external things, such as walking footsteps, soft dialogue, and multimedia sound. I think there is only music here, and only text in the point of view is too simple. If there are more elements, my sense of immersion would be stronger | | | | | | | |
| **4. Navigation**   1. Do you have any trouble finding directions or exits?   我觉得有点困难，主要是在方向上。我觉得我会搞不太清楚我下一步该看哪里了，因为画面旋转之后，我有点分不清方向。  I find it a little difficult, mainly in the direction. I don't think I'm going to know exactly where I'm going to look next, because after the rotation, I'm a little confused.   1. Do you know where you are in the exhibition?   我大概知道的，这个不难。你看一圈之后就大概知道。  I have a general idea. You'll get the idea after you look around. | | | | | | | |
| **5. Learning**   1. Do you think you can learn anything from this exhibition?   跟学习知识比，我感觉我是能了解一些信息，这个博物馆对我来讲还是太普通了。  Compared with learning knowledge, I feel that I can learn some information. This museum is still too common for me.   1. Did you find the exhibition interesting?   我觉得挺有趣的，这种全景游览的形式挺有趣的。有时候我没空出去玩，我可能会先看看这个，或许可以帮我决定下次去哪里。  I think it's kind of interesting, it's kind of interesting to have this way of panoramic tour. Sometimes I don't have time to go out, I might look at this first, maybe it can help me decide where to go next time. | | | | | | | |

| NO. B9 | Gender | Age | | | | | Time：3.8 |
| --- | --- | --- | --- | --- | --- | --- | --- |
|  | Female Male√ | ≤19 | 20-29√ | 30-39√ | 40-49 | ≥50 |  |
| **1.The overall feeling**   1. How did you feel about the experience?   我感觉不错，我喜欢这种体验。  I feel good, and I like the experience   1. What were some of the problems you encountered?   没有，感觉好像没什么问题，我觉得展览可能有点小，也有点简单吧。  No, it doesn't seem to be a problem. I think the exhibition might be a little small and a little simple. | | | | | | | |
| **2. Authenticity**   1. A：Do you feel real?   我感觉还是挺真实的，我觉得这个图像处理的很好，空间感和光线感都不错。  I think it's very realistic. I think the image is very well processed. The sense of space and light is very good.   1. Does it feel like visiting a real museum exhibition?   我觉得挺像的，而且环境很美，这个是我喜欢的地方。  Yes，I think it is. The environment is beautiful, and this is my favorite. | | | | | | | |
| **3. Interactivity**   1. How do you feel about interacting with the virtual exhibition?   互动吗，我觉得好像没什么互动。毕竟是博物馆吧，博物馆的展品你只能看看就好了，也不能碰他们。  Interaction? I don't think there's any interaction. After all, it's a museum. You can only look at the artifacts in a museum, and you can't touch them.   1. How do you feel the virtual exhibition respond to you?   虚拟博物馆的回应吗？我觉得反应很常规，没什么特别的地方。比如你想看看展品，也就是静态在那展示给你看。  Virtual museum response? I thought it was normal, nothing special. Let's say you want to look at the exhibits, which are still there for you to see. | | | | | | | |
| **4. Navigation**   1. Do you have any trouble finding directions or exits?   还好吧，我们感觉到有什么太大困难。  It's okay. We're feeling a little bit of trouble.   1. Do you know where you are in the exhibition?   知道的，我感觉展览没那么复杂，还是可以知道自己的位置。  Yes, I feel that the exhibition is not so complicated, you can still know where you are. | | | | | | | |
| **5. Learning**   1. Do you think you can learn anything from this exhibition?   我觉得多少学到一些。这些展览我虽然不是都能看懂，但是里面有很多我以前不知道的信息，比如我没想到古代人也会制作建筑模型，而且还很精致。  I think I've learned something. I didn't understand all the exhibits, but there was a lot of information in them that I didn't know before. For example, I didn't think that ancient people could make architectural models, and they were very exquisite.   1. Did you find the exhibition interesting?   还是挺有趣的，古代的图纸和模型我以前都没看过，这是我第一次接触这样的东西。  It was interesting. I had never seen any ancient drawings or models before, so this was the first time I had ever seen something like this. | | | | | | | |

| NO. C5 | Gender | Age | | | | | Time：  3.8 |
| --- | --- | --- | --- | --- | --- | --- | --- |
|  | Female√ Male | ≤19 | 20-29 | 30-39√ | 40-49 | ≥50 |  |
| **1.The overall feeling**   1. How did you feel about the experience?   我挺喜欢的，这还蛮有意思的。  I like it. It's kind of fun.   1. What were some of the problems you encountered?   还好，我没遇到什么大问题。  I didn't have any big problems. | | | | | | | |
| **2. Authenticity**   1. A：Do you feel real?   嗯，挺真实的，画面很清晰很真实。  Yes, it's very real. The graphic is very clear and very real.   1. Does it feel like visiting a real museum exhibition?   还是有一点像在真实的博物馆的。  Yes, it's a bit like being in a real museum.  Further question: 为什么说是“有一点”？Why "a bit"?  这个画面很真实，我第一次打开的时候确实感觉很美，很真实。但是游览或者游走的感觉又不是很好。我不知道如何描述那种感觉，就是游走的感觉有一点不真实。  This graphic is very real, the first time I opened it did feel beautiful, very real. But the feeling of visiting or walking is not very good. I don't know how to describe it, but it's a little unreal to walking in the virtual space. | | | | | | | |
| **3. Interactivity**   1. How do you feel about interacting with the virtual exhibition?   有互动，但是太简单了。这种网上的展览，其实应该加点更有趣的互动，比如动画或者影像。  There's interaction, but it's too simple. This kind of online exhibition, in fact, should add more interesting interaction, such as animation or video.   1. How do you feel the virtual exhibition respond to you?   有回应，还是跟刚才说的一样，太简单了  There's a response. Again, that's too simple. | | | | | | | |
| **4. Navigation**   1. Do you have any trouble finding directions or exits?   这是有点困难。我觉得这个展览最麻烦的地方，就是每次切换场景，要重新找到自己的看展的方向，这真是很麻烦。  It's a little difficult. I think the most troublesome part of this exhibition is that every time you switch scenes, you must find your direction to see the exhibition, which is really troublesome.   1. Do you know where you are in the exhibition?   是的，我大概知道自己在什么位置。  Yes, I know roughly where I am. | | | | | | | |
| **5. Learning**   1. Do you think you can learn anything from this exhibition?   我了解了一些知识吧，这个展览的信息还是很简单明确的。  I know something about it. The information of the exhibition is simple and clear.   1. Did you find the exhibition interesting?   我觉得还好，这个展览太简单了，就像我之前说的，我想这个展览可以提供更多形式的知识，比如不光是图文。因为每个人需求不一样，我更喜欢看视频。  I think it's OK, this exhibition is too simple, as I said before, I think this exhibition can provide more forms of knowledge, such as not only pictures and texts. Because everyone has different needs, I prefer to watch videos. | | | | | | | |

| NO.C6 | Gender | Age | | | | | Time：  3.9 |
| --- | --- | --- | --- | --- | --- | --- | --- |
|  | Female√ Male | ≤19 | 20-29 | 30-39√ | 40-49 | ≥50 |  |
| **1.The overall feeling**   1. How did you feel about the experience?   我觉得这个还不错，我没有用过这种，这比我想象的有意思。  I think it's a good one. I haven't used it before. It's more fun than I thought.   1. What were some of the problems you encountered?   我目前还好，我没有发现特别严重的问题。但是有些小问题我不太喜欢，比如为什么模型的给的视角那么少，我想仔细看看。  I'm fine so far. I don't see any serious problems. But there are some small problems I don't like, such as why the model gives so few perspectives, I want to have a closer look. | | | | | | | |
| **2. Authenticity**   1. A：Do you feel real?   嗯，这种虚拟博物馆的感觉还是很真实的  Yes, the virtual museum feel is very real   1. Does it feel like visiting a real museum exhibition?   是的，这是很像博物馆的感觉。这个空间感非常好，我觉得我有在一个建筑里的感觉。特别是当你发现你可以放大很多细节的时候，你会觉得更像在真的博物馆里。  Yeah, it's very museum-like and the sense of space is very good, and I feel like I'm in a building especially when you find that you can zoom in on a lot of details, you feel more like you're in a real museum. | | | | | | | |
| **3. Interactivity**   1. How do you feel about interacting with the virtual exhibition?   我觉得还可以，我想要看展品的信息，我点击展品的小蓝点，它们都会提供。我想去哪里，也基本都能去。虽然不能到任何地方，但是我可以放大。唯一的问题是我没法跟展品有太多的互动，可以看到视角太少。  I think it's OK, I want to see the information on the exhibits, I click on the little blue dots on the exhibits, they all show me some information. I can go pretty much anywhere I want. Although I can't go anywhere, but I can zoom in and the only problem is that I can't interact with the exhibits too much, can I see too little perspective   1. How do you feel the virtual exhibition respond to you?   还好吧，我觉得唯一的问题我刚才也说了，我没法看太多视角。  It's okay. I think the only problem I have as I said is that I can't see too many angles. | | | | | | | |
| **4. Navigation**   1. Do you have any trouble finding directions or exits?   方向有点不太随心所欲，我不知道这是不是我自己的问题，当我想去一个方向的时候，他总是产生让我意外的结果。尽管如此，大部分时候还好。  I don't know if it's my own fault, but when I try to go in one direction, it always surprises me. Most of the time, though, it's fine.   1. Do you know where you are in the exhibition?   我大概知道自己的位置。  I know roughly where I stand. | | | | | | | |
| **5. Learning**   1. Do you think you can learn anything from this exhibition?   我觉得能学到东西，他们展品很多，仔细看的话可以看到很多有意思的信息。  I think you can learn something. They have a lot of exhibits, and if you look closely you can see a lot of interesting information.   1. Did you find the exhibition interesting?   我觉得我这个展览有意思，但是模型太简单啦，我想看得更多些。  I thought my show was interesting, but the models were too simple. I wanted to see more. | | | | | | | |

| NO.B10 | Gender | Age | | | | | Time：  3.10 |
| --- | --- | --- | --- | --- | --- | --- | --- |
|  | Female Male√ | ≤19 | 20-29√ | 30-39 | 40-49 | ≥50 |  |
| **1.The overall feeling**   1. How did you feel about the experience?   还不赖，我感觉还不错，这是看起来很新颖的技术  Not bad. I feel good about it. It's a novel technology.   1. What were some of the problems you encountered?   我觉得有的展品上的字我看不太清楚，我记得是一个白色的展板，背景太亮，文字都看不到了。  I think I can't see the words on some exhibits very clearly. I remember it was a white display board, and the background was so bright that the words could not be seen. | | | | | | | |
| **2. Authenticity**   1. A：Do you feel real?   是的，我觉得比较真实。  Yes, I think it's real.   1. Does it feel like visiting a real museum exhibition?   我有这种感觉，就是在真的博物馆中看的感觉。  I have this feeling. I have this feeling in a real museum. | | | | | | | |
| **3. Interactivity**   1. How do you feel about interacting with the virtual exhibition?   互动的话，我觉得互动不太多。我基本都是以看为主，我感觉好像没有什么互动的感觉。  I don't think there's much interaction. I basically focus on watching, and I don't feel like I have any interactive feeling.   1. How do you feel the virtual exhibition respond to you?   切换场景和点击信息的反应还比较灵敏，反应还算不错。  Switching scenes and click on the information of the response is relatively sensitive. Response is not bad. | | | | | | | |
| **4. Navigation**   1. Do you have any trouble finding directions or exits?   我好像没有印象我在这方面有遇到麻烦，还不错。  I don't seem to have the impression that I'm having any trouble with this. It's not bad.   1. Do you know where you are in the exhibition?   我觉得我好像知道。  I feel like I know. | | | | | | | |
| **5. Learning**   1. Do you think you can learn anything from this exhibition?   嗯，我觉得又学到一些，很多东西我是头一回见。  Yes, I think I've learned something, a lot of things I've never seen before.   1. Did you find the exhibition interesting?   这个展览整体感觉还是不错的，这是很新颖的展览。尤其是空间感和真实感很强烈，而且展示的东西也很有趣。我觉得这个展览是有趣的。  The overall feeling of the exhibition is good, it is a very novel exhibition. Especially the sense of space and reality is very strong, and the things on display are very interesting. I found the exhibition interesting. | | | | | | | |

| NO.C7 | Gender | Age | | | | | Time：  3.11 |
| --- | --- | --- | --- | --- | --- | --- | --- |
|  | Female√ Male | ≤19 | 20-29 | 30-39√ | 40-49 | ≥50 |  |
| **1.The overall feeling**   1. How did you feel about the experience?   还不错，我没用到过这种，这个挺简单的，不用戴眼镜就可以看，就是那种很大的眼镜。  It's not bad. I haven't used it before. It's easy to use without glasses, you know, big glasses.  Further question：是VR眼镜吗？VR glasses?  是的，我在商场里用过，不过这个展览没有VR效果那么3d就是了，但是这个更方便简单。  Yes, I used it in the mall, but this exhibition is not as 3D as VR, but this is more convenient and easier.   1. What were some of the problems you encountered?   还好我觉得我还没遇到什么问题。  Fortunately, I don't think I've had any problems yet. | | | | | | | |
| **2. Authenticity**   1. A：Do you feel real?   嗯，我觉得这个还不错，就是感觉有点生硬，没那么VR那么有3D 感。  Yes, I think this one is good, but it feels a little stiff, not as 3D as VR.   1. Does it feel like visiting a real museum exhibition?   我觉得还是很像真实博物馆，这很像一种动态的照片，介于3dVR和2d照片之间的感觉。  I think it's still very much like a real museum. It's very much like a moving photograph, which between a 3DVR and a 2D photograph. | | | | | | | |
| **3. Interactivity**   1. How do you feel about interacting with the virtual exhibition?   互动不是很多，展品都是很死板的摆放在那里。我觉得既然是网上的展览，我想可以有更多的互动。我是喜欢有人给我讲解，这里可以设计一个讲解员讲解我会更有耐心看下去。  There's not a lot of interaction, and the exhibits are very rigid. I think since it's an online exhibition, I think there can be more interaction. I do like people to explain to me, I can design a narrator here and I will be more patient to watch.   1. How do you feel the virtual exhibition respond to you?   回应的比较简单吧。  The response is simple. | | | | | | | |
| **4. Navigation**   1. Do you have any trouble finding directions or exits?   嗯，有遇到过，我觉得我没法完全弄懂这个看展的路线，它会到处跑，而不是我按照我想的方向前进，这个很奇怪。  Yes, I did. I didn't think I could quite understand the route of the exhibition. It would go everywhere instead of going in the direction I wanted to go, which was very strange.   1. Do you know where you are in the exhibition?   我好像大概知道自己的位置，但是不是很确切吧。  I sort of know where I am, but I'm not quite sure. | | | | | | | |
| **5. Learning**   1. Do you think you can learn anything from this exhibition?   我没什么感觉，我记不得我看的具体内容了。我记得一些展板和模型，可能我不是很感兴趣吧。  I don't feel anything. I can't remember exactly what I read. I remember some boards and models, maybe I'm not very interested.   1. Did you find the exhibition interesting?   还行吧，我觉得不用眼镜就能体验VR类似的体验，这件事本身很有趣。  Not bad, I think it's interesting to be able to experience something similar in VR without VR glasses. | | | | | | | |

| NO. C8 | Gender | Age | | | | | Time：  3.11 |
| --- | --- | --- | --- | --- | --- | --- | --- |
|  | Female√ Male | ≤19 | 20-29 | 30-39√ | 40-49 | ≥50 |  |
| **1.The overall feeling**   1. How did you feel about the experience?   我感觉一般吧,但是这个展览还是不错的。  Not bad, and this exhibition itself is good.   1. What were some of the problems you encountered?   我觉得场景切换的时候让我不是很自然，尤其在边缘出现那种扭曲让我感觉不好，而且我经常找不到方向，让我觉得很混乱。  I don't think it's natural for me to switch scenes, especially when there's that kind of twist at the edges that makes me feel bad, and I often can't find the direction, which makes me feel confused. | | | | | | | |
| **2. Authenticity**   1. A：Do you feel real?   真实感还好，看起来还是蛮真实的。  It's real. It looks real.   1. Does it feel like visiting a real museum exhibition?   嗯，还不错啊，这感觉是在真实的博物馆里面。这个图像本来就是真实的吧，不是虚拟的3d模型。  Yes, that's good. It's like being in a real museum. I think it’s made by real images, not a virtual 3D model. | | | | | | | |
| **3. Interactivity**   1. How do you feel about interacting with the virtual exhibition?   我觉得互动不是很好吧，因为好像也没有什么有趣的互动。  I don't think the interaction is very good, because there doesn't seem to be any interesting interaction.  Further question：你能具体说下你期待的互动吗？Can you be specific about the interesting interaction you expect?  我想知道某个展品的的故事，谁用过它，发生了什么，但是展品给我的信息只有那么一些。  I wanted to know the story of an object, who used it, what happened to it, but the object gave me only so much information.   1. How do you feel the virtual exhibition respond to you?   回应的话，嗯，我觉得一般般吧，我感觉我的行为没有得到很多的回应，都是一些基础的展示，让我一开始觉得很有意思，实际的感觉倒是一般。  In response, well, I think it's just so-so, I feel like I didn't get a lot of response to my behavior, it was just some basic presentation that I found interesting at first, but actually it was so-so. | | | | | | | |
| **4. Navigation**   1. Do you have any trouble finding directions or exits?   我觉得方向有点乱，我每次都得重新确认前进的箭头，而且有几处我试了好几次，不太好用。  I thought the direction was a bit confusing, I had to reconfirm the forward arrow each time, and there were a few locations that I tried several times and didn't work very well.   1. Do you know where you are in the exhibition?   我觉得一般，不是很明确。  I think it's not very clear. | | | | | | | |
| **5. Learning**   1. Do you think you can learn anything from this exhibition?   这要看怎么定义学习了，我觉得如果你只是想了解一点信息，那你就学到了；但是，如果你想知道更多信息，我觉得这些还不够，因为信息太简单了。我想知道更多一些，这是故宫的展览，应该有很多故事。  It depends on how to define the learning. I think if you just want a little information, you've learned; However, if you want to know more information, I don't think this is enough, because the information is too simple. I want to know more, this is the exhibition of the Palace Museum, should have a lot of stories.   1. Did you find the exhibition interesting?   我觉得还行，展品很丰富，但是展品的信息太简单了，要是更多点信息就好了。  I think it's OK. The artifacts are rich, but the information on the exhibits is too simple. I wish I had more information. | | | | | | | |

| NO. B11 | Gender | Age | | | | | Time：  3.11 |
| --- | --- | --- | --- | --- | --- | --- | --- |
|  | Female Male√ | ≤19 | 20-29√ | 30-39 | 40-49 | ≥50 |  |
| **1.The overall feeling**   1. How did you feel about the experience?   我感觉挺好的。  I feel fine.   1. What were some of the problems you encountered?   到没什么大的问题。如果非要说一个问题的话，就是没去过现场的话方位可能有问题。漫游好像缺少方向性，它就有一个点，如果我点的话就会走过去。但是如果我没去过现场，我可能不知道自己在什么方向。  It's no big problem. If I have to say one thing, I don't know if I've ever been to the site. Roaming seems to lack direction, it has a point, if I click it will be walking. But if I haven't been there, I might not know where I'm going. | | | | | | | |
| **2. Authenticity**   1. A：Do you feel real?   这个挺真实的。  That's real.   1. Does it feel like visiting a real museum exhibition?   如果和VR的效果一样的话就更好一些。现在这个展览，可能是因为这个屏幕的限制，我觉得还是不够真实。  It would be better if it had the same effect as VR. Now this exhibition, maybe because of the limitations of this screen, I think is still not realistic enough.  Further question:这个不真实的感觉只是屏幕导致的吗？Is this unreal feeling just caused by the screen?  不只是屏幕，还因为这些展品没法触摸，就缺少让我身临其境的元素。  Not only the screen, but because the exhibits are untouchable, they lack the elements to immerse me. | | | | | | | |
| **3. Interactivity**   1. How do you feel about interacting with the virtual exhibition?   互动的感觉一般，如果能像VR一样戴上VR眼镜的话，效果会更好些。  The interactive feeling is mediocre, and if you can wear VR glasses like VR, the effect will be even better.  Further question:你是指沉浸感吗？Do you mean immersive?  对。Yes.   1. How do you feel the virtual exhibition respond to you?   回应的话，我觉得还行。如果没去过这个展览，这个展览对我反应让我了解一些。  In response, I think it's OK. If you haven't been to this exhibition, the reaction to this exhibition tells me something. | | | | | | | |
| **4. Navigation**   1. Do you have any trouble finding directions or exits?   我觉得不是很好，我觉得这个展览对方向上缺乏明确的指引，你会搞不清楚方向。这个问题尤其是对没去过现场的人更为严重。我在观展的过程中，能感觉到被这个系统牵引前进，但是我自己不知道自己身居何处。  I don't think it's very good. I think the exhibition lacks clear directions to the direction of the other side, so you won't know the direction. This problem is especially serious for people who have not been to the scene. As I watched the exhibition, I could feel pulled by the system, but I had no idea where I was.  Further question:底下有个小地图你有发现吗？There's a little map at the bottom. Did you see it?  那个小地图我有看到，但是对地图不敏感的人可能也搞不清楚方向。  I have seen the small map, but people who are not sensitive to maps may not be able to figure out the directions.   1. Do you know where you are in the exhibition?   有些时候知道，有些时候不行。因为提供的点位缺乏方向，所以我会迷失方向。  Sometimes I did, sometimes I didn’t. get lost because of the lack of direction provided by the points. | | | | | | | |
| **5. Learning**   1. Do you think you can learn anything from this exhibition?   有的，我有学习到。Yes, I have learned.   1. Did you find the exhibition interesting?   嗯，挺有趣的Well, it's interesting  Further question: 你能具体说说吗？Can you tell me more?  我看了一些我平常看不到的东西。通过这个展览我对这个展示主题有一定的了解。但是，我觉得如歌一个人的文化底蕴不足的话，就可能不知道在看什么。里面的小点我有点开，但是只有一点文字，很快我就嫌麻烦不想打开了。我觉得这个要有自动播放的语言导览就好了。  I saw something I don't normally see. Through this exhibition, I have a certain understanding of the exhibition theme. However, I think if a person's cultural background is insufficient, he may not know what he is looking at. I opened the dot, but there was only a little text, and soon I didn't bother to open it. I think it would be nice to have an auto-play language guide. | | | | | | | |

| NO.E2 | Gender | Age | | | | | Time：  3.13 |
| --- | --- | --- | --- | --- | --- | --- | --- |
|  | Female Male√ | ≤19 | 20-29 | 30-39 | 40-49 | ≥50√ |  |
| **1.The overall feeling**   1. How did you feel about the experience?   这很壮观。  It's spectacular.  Further question:您能说详细点吗？Could you tell me more?  这个展览体现了我们的祖先想象力很丰富，很精美。  This exhibition shows that our ancestors were very imaginative and exquisite.   1. What were some of the problems you encountered?   上楼梯时候没有指示，这让我不知道怎么上楼梯，怎么下楼梯。  There were no instructions to go up the stairs, so I didn't know how to go up the stairs or down the stairs. | | | | | | | |
| **2. Authenticity**   1. Do you feel real?   还比较真实，我去过故宫，看过类似的展览，这个感觉很真实。  It's real. I've been to the Forbidden City and seen similar exhibitions. It feels real.   1. Does it feel like visiting a real museum exhibition?   有点不太像。  Not quite。  Further question:您能说详细点吗？Could you tell me more?  这个展品有点像壁画一样，让我有点像是在看一幅幅画。这让我感觉死板，也缺少空间感和立体感。  This exhibit is something like murals, which makes me a bit like looking at one painting after another. It makes me feel rigid, but also lack of space and three-dimensional sense. | | | | | | | |
| **3. Interactivity**   1. How do you feel about interacting with the virtual exhibition?   我感觉一般。  I feel just so.  Further question:您能说详细点吗？Could you tell me more?  这不是理想中的互动，比如上楼梯。还有找那个展品过程不顺利。  It's not the ideal interaction, like going up the stairs. And the search for the artifact didn't go well.   1. How do you feel the virtual exhibition respond to you?   展览对我反应不是很灵活，可能我不经常用电脑，比如画面旋转起来不太顺手。  The exhibition is not very flexible to me, maybe I do not often use the computer, such as the rotation of the view is not very smooth. | | | | | | | |
| **4. Navigation**   1. Do you have any trouble finding directions or exits?   我觉得有困难。有时候那个箭头点击的时候，好像并不总是我想去的方向。比如上楼或下楼的时候。我想最好是我点哪里，它就到哪里。  I find it difficult. Sometimes when that arrow clicks, it doesn't always seem to go where I want to go. Like when you go upstairs or downstairs. I think it's better if it goes where I order it.   1. Do you know where you are in the exhibition?   我不是很清楚，我对自己的位置概念比较模糊。我就关注展品，没太多想自己在什么位置。  I'm not sure. I have a vague idea of where I am. I just focused on the exhibits and didn't think too much about where I was. | | | | | | | |
| **5. Learning**   1. Do you think you can learn anything from this exhibition?   我对建筑设计不太了解，我就看看表面的，我学到一些传统的纹样和图形，比如有些动物，我知道古代权贵的建筑用这些动物装饰。我以前去过山西传统的民居，都是当地有权有势的人家也会有这些装饰。  I didn't know much about architectural design, so I just looked at the artifacts surfacely. I learned some traditional patterns, such as some animals. I have been to traditional houses in Shanxi before, and I knew that the buildings of the ancient power and nobility used these animals to decorate the traditional dwellings in Shanxi.   1. Did you find the exhibition interesting?   我觉得挺有意思的，尤其是色彩搭配给我很舒服的感觉。  I think it's very interesting, especially the color matching gives me a very comfortable feeling. | | | | | | | |

| NO.C9 | Gender | Age | | | | | Time：  3.13 |
| --- | --- | --- | --- | --- | --- | --- | --- |
|  | Female Male√ | ≤19 | 20-29 | 30-39√ | 40-49 | ≥50 |  |
| **1.The overall feeling**   1. How did you feel about the experience?   我挺喜欢这个展览，我感觉还不错。  I like the exhibition. I feel good about it.   1. What were some of the problems you encountered?   我可能在操作上有点问题。不知道为什么我，我总是感觉不是很顺利，就是它的视野总是和我预期的不一致。而且还有点拉伸的变形感，这个我也感觉不好。  I may have a problem with the operation. I don't know why, I always feel it is not very smooth, is that its vision is always not consistent with my expectation. And there's a little bit of stretch and deformation, which I don't feel good about either. | | | | | | | |
| **2. Authenticity**   1. Do you feel real?   还不错的，我觉得还原的挺真实的。  It's not bad. I think it's real.   1. Does it feel like visiting a real museum exhibition?   还行，我觉得和真实的博物馆有点像。但是它们差别也很明显，比如画面会变形，这和真实博物馆有差别。此外，看到的就是在这个博物馆的里的行为都不是很自然，没法像真博物馆一样随心所欲。  Not bad. I think it's kind of like a real museum. But the differences are also obvious, such as the deformation of the image, which is different from the real museum. In addition, what we can see is that the behavior in this museum is not very natural and can not be as arbitrary as the real museum. | | | | | | | |
| **3. Interactivity**   1. How do you feel about interacting with the virtual exhibition?   互动我觉得不是很明显。好像我的行为都是被设定好了，这个互动不够自然。  I don't think the interaction is obvious. It's like my behavior is programmed. It's not a natural interaction.   1. How do you feel the virtual exhibition respond to you?   回应不够好，还是我刚才说的，感觉我的行为都是被设定好了。除了可以旋转视野，我好像没什么可选的互动。  The response was not perfect, and again, as I said, it felt like my behavior was programmed. Other than being able to rotate the view, I don't seem to have a choice of interactions. | | | | | | | |
| **4. Navigation**   1. Do you have any trouble finding directions or exits?   我觉得有点小问题，我感觉因为这个程序把一切设定好了，所以我想要去的方向，它不会呈现给我。它呈现的视野都是程序安排好的，这个不自然。不过当你慢慢习惯这个操作之后，会好些。  I think it's a little bit of a problem, I feel like because it's programmed everything, so I want to go in the direction, it's not going to give me the view that it's programmed in, it's not natural but as you get used to it, it's going to get better.   1. Do you know where you are in the exhibition?   不太清楚，我觉得比较混乱。这可能还是和视野不够自然有关系吧。  I don't know. I think it's confusing. This may still have something to do with the lack of natural vision. | | | | | | | |
| **5. Learning**   1. Do you think you can learn anything from this exhibition?   嗯，我觉得展览可以这么做，可以让人不需要到博物馆里面就能看到很多不常见的文物。  Yes, I think exhibitions can do that. They can give people a chance to see a lot of unusual objects without going to a museum.   1. Did you find the exhibition interesting?   还不错的，我觉得还是很有意思的。这个虽然用起来有点操作的问题，但是我觉得一旦用习惯也就还好。  It's not bad. I think it's interesting. Although this is a bit of an operation problem, but I think once used it will be OK. | | | | | | | |

| NO. C10 | Gender | Age | | | | | Time：  3.14 |
| --- | --- | --- | --- | --- | --- | --- | --- |
|  | Female Male√ | ≤19 | 20-29 | 30-39√ | 40-49 | ≥50 |  |
| **1.The overall feeling**   1. How did you feel about the experience?   感觉还不错。  Not bad.   1. What were some of the problems you encountered?   我好像没遇到什么太大问题，不过我觉得我不是很熟练地使用这个程序，有些地方我不是很会用，比如我经常会找不到下一步的箭头。  I don't seem to be having any major problems with it, but I don't think I'm very skillful with it. There are some things I'm not very good at, like I often can't find the next arrow. | | | | | | | |
| **2. Authenticity**   1. Do you feel real?   真实的，我觉得比较真实，尤其这个画面给我的感觉很真实。  I think more real, especially frames gives me the feeling is very real.   1. Does it feel like visiting a real museum exhibition?   有一点像，但是有时候我很难在这个空间中自由走动，不过比真实的博物馆好的地方，是没有很多游客会干扰你看展品，也避免了人可能损毁展品。  A little bit like a real museum, but sometimes it's hard for me to move around freely in this space, but what's better than a real museum is that you don't have a lot of visitors to disturb your view of the artifacts, and you don't have people to damage the exhibits. | | | | | | | |
| **3. Interactivity**   1. How do you feel about interacting with the virtual exhibition?   没什么太多互动吧，就是简单的看展。  Not much interaction, just a simple exhibition.   1. How do you feel the virtual exhibition respond to you?   我觉得这种回应很简单。我其实也看过很多跟故宫建筑有关的东西，也了解一些相关的知识，比如屋脊上的神兽，所以我能大概知道自己在看什么。但是这个展览给我们的展示的信息太少了，我觉得大多数人看不懂。  I think it's a simple response. I've seen a lot of things about the Forbidden City, and I know a lot about it, like the mythological animal on the roof, so I have a pretty good idea of what I'm looking at. But the exhibition gives us so little information that I don't think most people can understand it. | | | | | | | |
| **4. Navigation**   1. Do you have any trouble finding directions or exits?   还行，这个展览的在这方面好像有点别扭，就是我常常看不出来我哪边去过了，哪边没去过，至少我觉得箭头最好能区分一下。这是让我感觉有点不太好用，可能我不太熟悉这种程序。  Not bad, this exhibition seems to be a little troublesome, and I often can't tell which side I have been to, at least I think it would be better to distinguish the arrows. This is a bit awkward for me, maybe I'm not familiar with this kind of program.   1. Do you know where you are in the exhibition?   嗯，我大概知道自己的在什么位置。我在里面绕了两圈，展览的空间不大。  Yes, I sort of know where I am. I walked around it twice, and there wasn't much room for the exhibition. | | | | | | | |
| **5. Learning**   1. Do you think you can learn anything from this exhibition?   能学到，这里的知识够丰富的。而且，我觉得这个展览很新奇，足够激起人们的好奇心。但是信息要是更简单一些就好了，这样会有更多人学到东西。  Yes, there's plenty of knowledge here. Also, I think the exhibition is novel enough to pique people's curiosity. But if only the message were simpler, more people would learn.   1. Did you find the exhibition interesting?   还不错，就是展览稍微单调了些，不过这种展示方式本身已经很有趣了。  It's not bad, but the exhibition is a little monotonous, but the presentation itself is interesting. | | | | | | | |

| NO. C11 | Gender | Age | | | | | Time：  3.15 |
| --- | --- | --- | --- | --- | --- | --- | --- |
|  | Female√ Male | ≤19 | 20-29 | 30-39√ | 40-49 | ≥50 |  |
| **1.The overall feeling**   1. How did you feel about the experience?   挺好的。  It's fine.   1. What were some of the problems you encountered?   我觉得还行。我不太看得懂这个展览，很多字我不会读，最好能标注一下。  I think it's fine. I can't understand this exhibition very well. I can't read many words. I'd better mark them. | | | | | | | |
| **2. Authenticity**   1. Do you feel real?   嗯，这个倒是很真实，  Yes, that's real   1. Does it feel like visiting a real museum exhibition?   嗯，有这种感觉，像在一个真实博物馆里。  Yes, it's like being in a real museum. | | | | | | | |
| **3. Interactivity**   1. How do you feel about interacting with the virtual exhibition?   互动我觉得不是很多，这些看起来更像是照片，而不像是三维的展览。  I don't think there's much interaction. These look more like photographs than 3d exhibitions.  Future question: 为什么说像照片呢？Why does it look like a photograph?  因为只有正对展品的角度比较好，边缘的都会变形，展品的角度也不会因为我的视角展现出更多了。  Because only the Angle directly facing the exhibits is better, and the edge will be deformed, and the Angle of the exhibits will not show more because of my different perspectives.   1. How do you feel the virtual exhibition respond to you?   还行吧，我觉得没有太多的回应。如果说是切换场景的回应，我觉得方向还不够清晰，我需要花点时间适应一下。  Not bad. I don't think there's been a lot of response. I don't think the direction is clear enough and I need to get used to it a little bit. | | | | | | | |
| **4. Navigation**   1. Do you have any trouble finding directions or exits?   我觉得有点麻烦，刚才我也说了，就是每次切换场景的时候，我觉得方向有点混乱，特别是找下楼梯的出口时候。  I think it's a little bit of a problem, as I said, that every time I switch scenes, I feel a little confused, especially finding the exit to go down the stairs.   1. Do you know where you are in the exhibition?   我大概知道自己的位置，但是有些地方我想进去，却绕了好几次，比如一楼里面的展区。  I had a general idea of where I was, but there were some places I tried to get in, but I walked around several times, like the exhibition area on the first floor. | | | | | | | |
| **5. Learning**   1. Do you think you can learn anything from this exhibition?   我觉得还行，为了找那个展品，我有认真看这些展览。展览的物品很多，但是信息很少也很专业，我是看不太懂，我觉得可以加点辅助的信息，比如对生僻字加拼音，还可以加入更多有趣的信息，比如视频。  I think it's OK. I've looked at the exhibitions carefully in order to find the artifact. There are a lot of articles in the exhibition, but the information is very small and professional, which I can't understand. I think we can add some auxiliary information, such as adding pinyin to rare characters, and add more interesting information, such as videos.   1. Did you find the exhibition interesting?   还不错，就像我刚才说的那样，我希望加点有趣的视频之类的。我蛮喜欢这个展览的方式，让我可以随时去看展览，这个看展览的方式很酷。  It's not bad. Like I said, I hope to add some interesting videos or something else. I really like the way the exhibition is, I can go to the exhibition whenever I want, it's a cool way to see the exhibition. | | | | | | | |

| NO.B12 | Gender | Age | | | | | Time：  3.15 |
| --- | --- | --- | --- | --- | --- | --- | --- |
|  | Female√ Male | ≤19 | 20-29√ | 30-39 | 40-49 | ≥50 |  |
| **1.The overall feeling**   1. How did you feel about the experience?   初步进入的整体感觉还可以，但是体验过一段时间就会觉得普通。  The overall feeling of the initial entry is OK, but after a period of experience will feel ordinary.   1. What were some of the problems you encountered?   参与感一般。  A sense of average participation.  Further question：Does it feel bad to participate?  是的，我感觉不太好，走路不太顺畅。此外相似的展品太多，（展品）没有特色化的提示，容易混淆。  Yes, I don't feel very well. I can't walk very smoothly. In addition, there are too many similar artifacts, and there are no specific hints, which is easy to be confused. | | | | | | | |
| **2. Authenticity**   1. Do you feel real?   还不错，真实度还可以。  It's not bad. It's real.   1. Does it feel like visiting a real museum exhibition?   在对于参观和学习知识的层面上，像在真实的博物馆。但是在操作上，没有临场的感觉的真实感。  On the level of visiting and learning knowledge, like in a real museum. But in operation, there is no sense of reality. | | | | | | | |
| **3. Interactivity**   1. How do you feel about interacting with the virtual exhibition?   总体还不错，尽管类似的相同的操作太多，但是整体感觉还是可以掌握的。  Overall not bad, although there are too many similar and identical operations, but the overall feeling is manageable.   1. How do you feel the virtual exhibition respond to you?   还行吧，就是这种反馈缺乏趣味度和吸引度，希望以后可以提升。  Not bad, but this kind of responses lacks interest and attraction. I hope it can be improved in the future. | | | | | | | |
| **4. Navigation**   1. Do you have any trouble finding directions or exits?   出口还好，但是在进入展区的时候，找方向上有困难。  The exit was fine, but I had difficulty finding my way into the exhibition area.  Further question：能具体说说吗Can you tell me more about it?  我有时候想进入一个展区会一直进不去，操作不顺利，希望这点以后可以优化。  Sometimes when I want to enter an exhibition area, I can't get in and the operation is not smooth. I hope this can be optimized in the future.   1. Do you know where you are in the exhibition?   大概知道，比如在一楼或二楼时候。  Probably, like on the first or second floor. | | | | | | | |
| **5. Learning**   1. Do you think you can learn anything from this exhibition?   我学习到整个博物馆的主题，和它展示的知识。我也简单了解了这个博物馆的风格。  I learned about the theme of the whole museum, and the knowledge it displayed. I also had a brief understanding of the style of the museum.   1. Did you find the exhibition interesting?   一般，我觉得知识性的学习比展览的趣味性更大。  In general, I find intellectual learning more interesting than exhibitions. | | | | | | | |

| NO. C12 | Gender | Age | | | | | Time：  3.15 |
| --- | --- | --- | --- | --- | --- | --- | --- |
|  | Female√ Male | ≤19 | 20-29 | 30-39√ | 40-49 | ≥50 |  |
| **1.The overall feeling**   1. How did you feel about the experience?   我觉得不赖，我喜欢这种体验。  I think it's not bad. I like the experience.   1. What were some of the problems you encountered?   我觉得还好，好像没有什么太大的问题。  I feel fine. There doesn't seem to be any big problem. | | | | | | | |
| **2. Authenticity**   1. Do you feel real?   嗯，我感觉还是蛮真实的。  Yes, it feels real to me.   1. Does it feel like visiting a real museum exhibition?   还是有点像在真的博物馆，就像在博物馆里看展品一样。当然我知道这只是虚拟的，我觉不可能完全跟真的博物馆一样。  It's still kind of like being in a real museum, like looking at artifacts in a museum. Of course, I knew it was only virtual, and I didn't think it could be exactly like a real museum. | | | | | | | |
| **3. Interactivity**   1. How do you feel about interacting with the virtual exhibition?   如果只是纯粹看展品，我觉得现在的互动足够了。  If you just look at the artifacts, I think the interaction is enough now.   1. How do you feel the virtual exhibition respond to you?   有互动的感觉，但是好像互动的水平比较低。  There is a sense of interaction, but it seems to be at a lower level.  Further question: 你能说的详细些吗？Could you tell me more about it?  我觉得展品是静态的，它的对我看的回应基本上就只能放大。其实这个展厅里大部分展品是图片和资料，这样放大倒没什么问题。但是那些立体的展品我是喜欢多个角度看，我觉得只能放大还不够。  In my opinion, the exhibits are static, and their response to my observation can only be enlarged. In fact, most of the exhibits in this exhibition hall are pictures and materials, so there is no problem in this way of responses. However, I like to view the 3d artifacts from multiple angles, so I think enlarging them is not enough. | | | | | | | |
| **4. Navigation**   1. Do you have any trouble finding directions or exits?   这个有点问题，找方向有点麻烦。我觉得这个设计有点问题，但是我能适应。  That's a little bit of a problem. I'm having trouble finding directions. I think there's something wrong with the design, but I can get used to it.   1. Do you know where you are in the exhibition?   我没有太注意到这个问题，现在回想起来，似乎大概有点乱。  I didn't pay much attention to it, and in retrospect, it seems like a bit of a mess. | | | | | | | |
| **5. Learning**   1. Do you think you can learn anything from this exhibition?   我觉得我学到一些，虽然我不是都能看得懂，但是有些展品的印象很深刻，比如那个屋脊上的小兽，以前我有了解一些，但是没这么近距离看过。我还看到一些我以前没注意到的地方，比如天花上的绘画。我以后在古建筑里，大概知道哪些是需要关注的重点了。  I think I have learned something, although I cannot understand all the exhibits, but some of the artifacts are very impressive, such as the small beast on the roof. I have known a little before, but after I have not seen it so closely. I also saw things I hadn't noticed before, like the paintings on the ceiling. In the future, I probably know which ones need to be paid attention to in an ancient building.   1. Did you find the exhibition interesting?   嗯，还行吧，谈不上有多有趣，但是展示的东西够多了，而且这种虚拟展览方式挺有意思。  It’s not too bad, not very interesting, but there are plenty of things on display, and the virtual exhibition is fun. | | | | | | | |

| NO. C13 | Gender | Age | | | | | Time：  3.15 |
| --- | --- | --- | --- | --- | --- | --- | --- |
|  | Female√ Male | ≤19 | 20-29 | 30-39√ | 40-49 | ≥50 |  |
| **1.The overall feeling**   1. How did you feel about the experience?   我觉得不赖，我喜欢这种体验。  I think it's not bad. I like the experience.   1. What were some of the problems you encountered?   我觉得还好，好像没有什么太大的问题。  I feel fine. There doesn't seem to be any big problem. | | | | | | | |
| **2. Authenticity**   1. Do you feel real?   嗯，我感觉还是蛮真实的。  Yes, it feels real to me.   1. Does it feel like visiting a real museum exhibition?   还是有点像在真的博物馆，就像在博物馆里看展品一样。当然我知道这只是虚拟的，我觉不可能完全跟真的博物馆一样。  It's still kind of like being in a real museum, like looking at artifacts in a museum. Of course, I knew it was only virtual, and I didn't think it could be exactly like a real museum. | | | | | | | |
| **3. Interactivity**   1. How do you feel about interacting with the virtual exhibition?   如果只是纯粹看展品，我觉得现在的互动足够了。  If you just look at the artifacts, I think the interaction is enough now.   1. How do you feel the virtual exhibition respond to you?   有互动的感觉，但是好像互动的水平比较低。  There is a sense of interaction, but it seems to be at a lower level.  Further question: 你能说的详细些吗？Could you tell me more about it?  我觉得展品是静态的，它的对我看的回应基本上就只能放大。其实这个展厅里大部分展品是图片和资料，这样放大倒没什么问题。但是那些立体的展品我是喜欢多个角度看，我觉得只能放大还不够。  In my opinion, the exhibits are static, and their response to my observation can only be enlarged. In fact, most of the exhibits in this exhibition hall are pictures and materials, so there is no problem in this way of responses. However, I like to view the 3d artifacts from multiple angles, so I think enlarging them is not enough. | | | | | | | |
| **4. Navigation**   1. Do you have any trouble finding directions or exits?   这个有点问题，找方向有点麻烦。我觉得这个设计有点问题，但是我能适应。  That's a little bit of a problem. I'm having trouble finding directions. I think there's something wrong with the design, but I can get used to it.   1. Do you know where you are in the exhibition?   我没有太注意到这个问题，现在回想起来，似乎大概有点乱。  I didn't pay much attention to it, and in retrospect, it seems like a bit of a mess. | | | | | | | |
| **5. Learning**   1. Do you think you can learn anything from this exhibition?   我觉得我学到一些，虽然我不是都能看得懂，但是有些展品的印象很深刻，比如那个屋脊上的小兽，以前我有了解一些，但是没这么近距离看过。我还看到一些我以前没注意到的地方，比如天花上的绘画。我以后在古建筑里，大概知道哪些是需要关注的重点了。  I think I have learned something, although I cannot understand all the exhibits, but some of the artifacts are very impressive, such as the small beast on the roof. I have known a little before, but after I have not seen it so closely. I also saw things I hadn't noticed before, like the paintings on the ceiling. In the future, I probably know which ones need to be paid attention to in an ancient building.   1. Did you find the exhibition interesting?   嗯，还行吧，谈不上有多有趣，但是展示的东西够多了，而且这种虚拟展览方式挺有意思。  It’s not too bad, not very interesting, but there are plenty of things on display, and the virtual exhibition is fun. | | | | | | | |

| NO. C14 | Gender | Age | | | | | Time：  3.15 |
| --- | --- | --- | --- | --- | --- | --- | --- |
|  | Female Male√ | ≤19 | 20-29 | 30-39√ | 40-49 | ≥50 |  |
| **1.The overall feeling**   1. How did you feel about the experience?   我觉得这还不错。  I think that's nice.   1. What were some of the problems you encountered?   我觉得方向指引不够明确，这影响我的感受。  I don't think the directions are clear enough, and that affects my experience. | | | | | | | |
| **2. Authenticity**   1. Do you feel real?   我感觉是真实的。  It's real.   1. Does it feel like visiting a real museum exhibition?   我觉得像，展示和风格上，都符合博物馆的氛围。  I feel like, in terms of presentation and style, it fits the atmosphere of a real museum. | | | | | | | |
| **3. Interactivity**   1. How do you feel about interacting with the virtual exhibition?   互动还不错，基本上能够了解这个展览所要表达的信息。  The interaction is quite good, and I can basically understand the message of the exhibition.   1. How do you feel the virtual exhibition respond to you?   回应还不错，但是交互性的回应有点少。  The response was good, but a little less interactive.  Further question: 能具体说说吗? Can you tell me more about it?  比如说要是有些交互式的导览就更好了，再加上能够触发式的互动就更有创意，让人可以保持最佳的观赏状态，而不会厌倦。  For example, some interactive tours would be better, and the interaction that can trigger is more creative, so that people can keep the best viewing state, and will not be bored. | | | | | | | |
| **4. Navigation**   1. Do you have any trouble finding directions or exits?   大方向还好，就是小方向上有点不太好掌控。比如看展品的视角方向，总是不很好的控制，似乎和行走的方向不一致。目前其他好像没有太大问题。  The general direction is fine, but the small direction is a little difficult to control. For example, the perspective direction of viewing exhibits is not always well controlled, which seems to be inconsistent with the direction of walking. Nothing else seems to be a problem now.   1. Do you know where you are in the exhibition?   我大概知道的。  I sort of know. | | | | | | | |
| **5. Learning**   1. Do you think you can learn anything from this exhibition?   我有学到东西，但是希望能够配上更多的大图，甚至加入一些动态图片，这样会更有吸引力。  I learned something, but I wish I could add more big pictures, maybe even some moving pictures, to make it more attractive.   1. Did you find the exhibition interesting?   趣味度还可以。  Not bad.  Further question: 能具体说说吗? Can you tell me more about it?  和在现场看不一样，能进入虚拟展览的话还是挺新鲜的。  It's something new to be able to enter a virtual exhibition, as opposed to seeing it live. | | | | | | | |

| NO.  C15 | Gender | Age | | | | | Time：  3.15 |
| --- | --- | --- | --- | --- | --- | --- | --- |
|  | Female√ Male | ≤19 | 20-29 | 30-39√ | 40-49 | ≥50 |  |
| **1.The overall feeling**   1. How did you feel about the experience?   我觉得一般，这个虚拟展览形式还是挺有意思的。  I think in general; the virtual exhibition format is quite interesting.   1. What were some of the problems you encountered?   好像没有特别大的问题，感觉展品太多，有点眼花缭乱。  There seems to be no big problem, feeling too many exhibits, a bit dazzling. | | | | | | | |
| **2. Authenticity**   1. Do you feel real?   还行，看起来还是非常真实的。这个应该是现场的照片制作的，还是蛮真实的。  Not bad. It still looks real. This is supposed to be a photo of the scene, or real.   1. Does it feel like visiting a real museum exhibition?   嗯，有些像真实的博物馆。但是展品标牌的字太小，我觉得看不太清楚。  Yes, it's kind of like a real museum. But the words on the display signs are too small for me to read clearly. | | | | | | | |
| **3. Interactivity**   1. How do you feel about interacting with the virtual exhibition?   我觉得互动上一般，但是对于一个传统的博物馆足够了。我觉得网上也就看看吧，如果想互动的话，我更愿意到现场看展。  I think the interaction is just so-so, but it's enough for a traditional museum. I think the online exhibition is just a look, and if I want to interact, I prefer to go to a live exhibition.   1. How do you feel the virtual exhibition respond to you?   嗯，反应一般，就像我刚才说的，对于参观一个普通的博物馆足够了。  The response was modest, as I said, enough for a normal museum visit.  Further question：不普通的博物馆是什么样子？What is an unnormal museum like?  现在很多博物馆有比较多的互动，比如放映互动影像啊，还有大型互动装置。我觉得这是个文物的历史博物馆，只要看看也差不多了。  Now many museums have more interactive, such as showing interactive videos, and large interactive installations. I think this is a historical museum of cultural relics. It's enough just to have a look. | | | | | | | |
| **4. Navigation**   1. Do you have any trouble finding directions or exits?   这个确实有点麻烦。我觉得那个导航的箭头没设计好，它前进的方向会和它指示的方向不一样，这点让我觉得有点麻烦。  This is a bit of a hassle. I think the navigation arrow is not well designed, it will go in a different direction than the direction it indicates, which makes me feel a bit troublesome.   1. Do you know where you are in the exhibition?   我按照指引的箭头走，没有考虑太多自己在哪里。不过我大概知道展览在一个两层楼里，并且我知道自己在哪一层。  I followed the arrow; I didn't think too much about where I was. But I probably knew it was in a two-story building, and I knew which floor I was on. | | | | | | | |
| **5. Learning**   1. Do you think you can learn anything from this exhibition?   能学到一些知识的，这个展览的展品很丰富。我也参观过一些历史博物馆，没这么多展品。  I can learn something, as the exhibits in this exhibition are very rich. I've also visited some history museums that don't have so many artifacts.   1. Did you find the exhibition interesting?   说到趣味性的话，我觉得展览主题本身很有专业性，所以也谈不上有趣吧。但是这种虚拟展示的感觉很有趣，我没体验过这种逛博物馆的形式。  Speaking of interesting I think the exhibition theme itself is very professional, so it is not interesting. But the feeling of virtual display is very interesting, I have never experienced this kind of museum form. | | | | | | | |

| NO. C16 | Gender | Age | | | | | Time：  3.15 |
| --- | --- | --- | --- | --- | --- | --- | --- |
|  | Female Male√ | ≤19 | 20-29 | 30-39√ | 40-49 | ≥50 |  |
| **1.The overall feeling**   1. How did you feel about the experience?   不错，我觉得这个展览的体验还不错，这个展示的效果也不错。  Yes, I think the experience of the exhibition is good, and the effect of the exhibition is good.   1. What were some of the problems you encountered?   好像没有什么明显的问题。  There doesn't seem to be any obvious problem. | | | | | | | |
| **2. Authenticity**   1. Do you feel real?   嗯，我觉得挺真实的。这个展示的效果挺好，画面感很好，空间感也不错。  Yes, I think it's real. The effect of the display is very good, the picture sense is good, the sense of space is also good.   1. Does it feel like visiting a real museum exhibition?   嗯，像的，挺像在博物馆里的感觉。  Yes, it's like being in a museum. | | | | | | | |
| **3. Interactivity**   1. How do you feel about interacting with the virtual exhibition?   说到互动的话，好像没有什么互动的选择吧。我觉得互动可以更多些，比如有个人物出来帮我讲解就好了，这个单纯看有点单调。  I don't think there are many options for interaction. I think the interaction can be more, such as a character to help me explain it, it is a little monotonous.   1. How do you feel the virtual exhibition respond to you?   回应有点单调。  The responses are a little monotonous. | | | | | | | |
| **4. Navigation**   1. Do you have any trouble finding directions or exits?   我觉得方向和出口上没什么太大问题。  I don't think there's much of a problem with the direction and exit.   1. Do you know where you are in the exhibition?   嗯，大致上是知道的。  Yes, roughly. | | | | | | | |
| **5. Learning**   1. Do you think you can learn anything from this exhibition?   还行，还是能学到一些东西吧。这个很适合老师远程授课，都不用带学生去现场了。  Not bad, still can learn something. This is very suitable for the teacher to teach remotely, and you don't have to take the students to the site.   1. Did you find the exhibition interesting?   我觉得这种历史性的展览谈不上多有趣吧，但是展览还是很特别的。  I don't think this kind of historical exhibition is very interesting, but it's still very special. | | | | | | | |

| NO. C17 | Gender | Age | | | | | Time：  3.15 |
| --- | --- | --- | --- | --- | --- | --- | --- |
|  | Female Male√ | ≤19 | 20-29 | 30-39√ | 40-49 | ≥50 |  |
| **1.The overall feeling**   1. How did you feel about the experience?   我觉得还不错，这种展览我从来没见过，很清晰，很漂亮的。  I think it's good. I've never seen this kind of exhibition before. It's very clear and beautiful.   1. What were some of the problems you encountered?   嗯，我觉得行走的过程有点顿挫，换面切换也不连贯，这可能是这种技术问题吧。  Well, I think the walking process is a little shaky, and the view switching is not coherent, which is probably a technical problem. | | | | | | | |
| **2. Authenticity**   1. Do you feel real?   是的，挺真实的。我觉得这个画面清晰度很高，让我觉得很有现场感。  Yes, quite real. I think the frames is very sharp and makes me feel very present.   1. Does it feel like visiting a real museum exhibition?   对，像在现场的感觉，但是在展厅里走动的时候会缺少现场的那种自由感。  Yeah, it's like being on site, but you don't have the freedom of being on site when you're walking around the exhibition. | | | | | | | |
| **3. Interactivity**   1. How do you feel about interacting with the virtual exhibition?   互动方面还行，我觉得这得看需求，比如我，我只需要看看就可以了，可能有人需要更多互动吧。  The interaction aspect is OK, I think it depends on the need, like me, I just need to look at it, maybe someone needs more interaction.   1. How do you feel the virtual exhibition respond to you?   我觉得有点不自然，反应有点机械，就是我每次点击下一步的时候，它切换的画面不是很自然，不过我慢慢习惯就好了。  I feel a little unnatural, the reaction is a little mechanical, is that every time I click next, it is not very natural to switch the view, but I'll get used to it. | | | | | | | |
| **4. Navigation**   1. Do you have any trouble finding directions or exits?   我觉得有点不太好用，我觉得方向上开始的时候让我有点困惑，后来慢慢熟悉这种规律就还好。  I think it is a little difficult to use. I think the direction of the beginning made me a little confused, and then gradually familiar with the law is OK.   1. Do you know where you are in the exhibition?   嗯，这个我大致上是知道的。  Yes, roughly. | | | | | | | |
| **5. Learning**   1. Do you think you can learn anything from this exhibition?   我觉得多少能学到一点的，它展示了一些基本信息。不过还是像我刚才说，这得看个人需求。  I think we can learn a little bit, and it shows me some basic information. But again, as I said, it depends on the individual.  Further question: 你能说的详细些吗？Could you tell me more about it?  假如你是专家的话或许想看到更多。不过专家会到现场看吧，这种线上只能看个大概。  If you're an expert you might want to see more. But the experts will come to the scene, and this kind of line can only be seen in general.   1. Did you find the exhibition interesting?   还是蛮有趣的，我挺喜欢看这样的展览，因为我不用去故宫也可以看（展览）。  It's interesting, and I like to see exhibitions like this, because I don't have to go to the Forbidden City to see it. | | | | | | | |
