# Supplementary material for: Evaluation of virtual tour in an online museum: Exhibition of Architecture of the Forbidden City
Source: PLoS One. 2022 Jan 6;17(1):e0261607. doi: 10.1371/journal.pone.0261607 (PMC8735558; doi:10.1371/journal.pone.0261607)
Supplement: S4 File — (DOCX) [file pone.0261607.s004.docx]

**Interview Guideline**

**Introduction**

- Name and background of the interviewer
- Purpose of the study
- Explain confidentiality and ask for consent of the interviewee

**General questions**

**-** **In this part, we'd like to know the users’ overall feelings about the experience.**

1. How did you feel about the experience?
2. What were some of the problems you encountered?

**Authenticity**

**-** **In this part, we'd like to know the users’ evaluation of the sense of reality of the EAFC, i.e., whether the EAFC's virtual space experience is similar to reality.**

1. Do you feel real?
2. Does it feel like visiting a real museum exhibition?

**Interactivity**

**-** **In this part, we'd like to know the users’ evaluation of the interactivity of the EAFC, i.e., whether the EAFC's responsive to users’ behavior as expected.**

1. How do you feel about interacting with the virtual exhibition?
2. How do you feel the virtual exhibition respond to you?

**Navigation**

**-** **In this part, we'd like to know the users’ evaluation the navigation of the EAFC, i.e., how well the user can identify direction and position in virtual space.**

1. Do you have any trouble finding directions or exits?
2. Do you know where you are in the exhibition?

**Learning**

**-** **In this part, we'd like to know the users’ evaluation of the learning of the EAFC, i.e., whether the user can get useful or interesting information from the EAFC.**

1. Do you think you can learn anything from this exhibition?
2. Did you find the exhibition interesting?

**Now we have come to the end of the interview, thank you so much!**
