# Supplementary material for: Evaluation of virtual tour in an online museum: Exhibition of Architecture of the Forbidden City
Source: PLoS One. 2022 Jan 6;17(1):e0261607. doi: 10.1371/journal.pone.0261607 (PMC8735558; doi:10.1371/journal.pone.0261607)
Supplement: S5 File — (DOCX) [file pone.0261607.s005.docx]

**Evaluation of Virtual Roaming in an Online Museum: Exhibition of Architecture of the Forbidden City**

**– COREQ Checklist**

| **No Item** | **Guide questions/description** | **Response** | **page no.** |
| --- | --- | --- | --- |
| **Domain 1: Research team and reflexivity** | | | |
| *Personal Characteristics* | | | |
| 1. Interviewer | Which author/s conducted the interview or focus group? | Jia Li | Title Page |
| 1. credentials | What were the researcher’s credentials?  E.g. PhD, MD | Jia Li: PhD student  Jin-Wei Nie: Ph D | Title Page |
| 1. occupation | What was their occupation at the time of the study? | Jia Li: PhD student  Jin-Wei Nie: A.P. | Title Page |
| 1. gender | Was the researcher male or female? | Jia Li, Jing Ye = male  Jin-Wei Nie = female | - |
| 1. experience and training | What experience or training did the researcher have? | All researchers had previous experience in interviewing and qualitative data analysis，and have received related academic training. | Title Page |
| 1. relationship established | Was a relationship established prior to study commencement? | No; the researcher did not know the participants they interviewed. | 16 |
| 1. participant knowledge of the interviewer | What did the participants know about the researcher? e.g. personal goals, reasons for doing the research | Participants were informed of the purpose of the study and the use of the data. | 12, Supporting Information |
| 1. Interviewer characteristics | What characteristics were reported about the interviewer/facilitator? e.g. Bias, assumptions, reasons and interests in the research topic | The researcher ensured that the questions were asked fairly and objectively, so as not to affect the participants during the interview. The participants were also encouraged to give feedback about their experiences as objective as possible, suggesting that this feedback could help improve the project. | Supporting Information |
| **Domain 2: study design** | | | |
| *Theoretical framework* | | | |
| 1. methodological orientation and theory | What methodological orientation was stated to underpin the study? e.g., grounded theory, discourse analysis, ethnography, phenomenology, content analysis | Semi-structured interview | 16 |
| 1. sampling | How were participants selected? e.g., purposive, convenience, consecutive, snowball | Stratified sampling | 16, table7 |
| 1. method o approach | How were participants approached? e.g. face-to-face, telephone, mail, email | Due to the COVID-19 pandemic; the interviews have been performed via Line or WeChat. | 6-7 |
| 1. sample size | How many participants were in the study? | 33 participants | 16 |
| 1. non-participation | How many people refused to participate or dropped out? Reasons? | No one declined to be interviewed. | - |
| 1. setting of data collection | Where was the data collected? e.g. home, clinic, workplace | Due to the COVID-19 pandemic, the researchers were at home and the interviews had been performed via Line and WeChat. | 5 |
| 1. presence of non-participants | Was anyone else present besides the participants and researchers? | To reduce distractions, researchers kept themselves alone in the room, and participants were asked to stay alone in the room as much as possible during the interview. | 16 |
| 1. description of sample | What are the important characteristics of the sample? e.g., demographic data, date | The sample was taken from ordinary people who had experienced the EAFC. | 12 |
| 1. interview guide | Were questions, prompts, guides provided by the authors? Was it pilot tested? | Yes; the study went through pilot test. | 13, Supporting Information |
| 1. repeat interviews | Were repeat interviews carried out? If yes, how many? | No | - |
| 1. audio/visual recording | Did the research use audio or visual recording to collect the data? | audio recording | 16 |
| 1. field notes | Were field notes made during and/or after the interview or focus group? | No | - |
| 1. duration | What was the duration of the interviews or focus group? | each interview lasted 15-30 minutes | 17 |
| 1. data saturation | Was data saturation discussed? | No | - |
| 1. transcripts returned | Were transcripts returned to participants for comment and/or correction? | No | - |
| **Domain 3: analysis and findings** | | | |
| *Data analysis* | | | |
| 24. Number of data coders | How many data coders coded the data? | One, Jia Li | - |
| 25. Description of the coding tree | Did authors provide a description of the coding tree? | Yes | 10-11 |
| 26. Derivation of themes | Were themes identified in advance or derived from the data? | The theme was identified in advance. | 10-11 |
| 27. Software | What software, if applicable, was used to manage the data? | SPSS | 11, 13 |
| 28. Participant checking | Did participants provide feedback on the findings? | No | - |
| *Reporting* | | | |
| 29. Quotations presented | Were participant quotations presented to illustrate the themes / findings? Was each quotation identified? e.g. participant number | No | - |
| 30. Data and findings consistent | Was there consistency between the data presented and the findings? | Yes | 19 |
| 31. Clarity of major themes | Were major themes clearly presented in the findings? | Yes | 19-24 |
| 32. Clarity of minor themes | Is there a description of diverse cases or discussion of minor themes? | Yes | 23-24 |
