# Supplementary material for: Evaluation of virtual tour in an online museum: Exhibition of Architecture of the Forbidden City
Source: PLoS One. 2022 Jan 6;17(1):e0261607. doi: 10.1371/journal.pone.0261607 (PMC8735558; doi:10.1371/journal.pone.0261607)
Supplement: Attachment — Submitted filename: Response to Reviewers-jiali0607.docx [file pone.0261607.s007.docx]

**Response to Reviewers**

**Reply to reviewer’s comments: Manuscript [PONE-D-21-07531]**

Dear Dr. Stefano Triberti,

We would like to thank the editor and reviewers for the invaluable comments and advice that helped improve our original manuscript. My partners and I studied the journal and reviewer's suggestions for revision carefully and revised the manuscript based on each suggestion. Please find our responses to the comments below.

**Editor Requirements**

1. **language editing by a native english speaker is still needed.**

Authors’ response: Thank you for your suggestion. We will continue to hand over the manuscript to a professional translation company for editing.

2. **the link to the online exhibition did not work at my first try; you should probably separate it from the final dot (without it, it works).**

Authors’ response: Thank you for your reminder. I have reedited this link to ensure its validity.

3. **Methodology lacks information, especially regarding the methodological approach for qualitative data.**

Authors’ response: Thank you for pointing out this problem. It was true that there was a lack of description in the methodology of the qualitative part of this study. We have strengthened this part in line 4-10 of p. 11 and lines 1–7 of p. 12 and added relevant references.

4. **Ethical approval was missing. This is necessary for consideration by PLOS ONE.**

Authors’ response: Thank you very much for your reminder. This paper did not provide an ethics statement, which was an oversight error on our part. We have added the ethical statements on p. 12, lines 19–21 and p. 13, lines1-3.

In accordance with IRB standards, the tests were carried out under the background that the participants fully understood the purpose, process, risk, and data use of the study. The participants filled in the questionnaire anonymously, and the data were recorded with their consent. This study adopts a paperless electronic questionnaire. Only with the consent of the participants, they can continue to fill in the questionnaire. The screenshot of the questionnaire home page is shown below.

The information in the figure above is as follows:

***valuation of Virtual Roaming in an Online Museum: Exhibition of Architecture of the Forbidden City***

*Thank you for taking the time to do this survey with us, please read the following instructions carefully before you begin.*

*1. With your help, we would to understand users' experience of virtual roaming of the online museum.*

*2. We want to find its weaknesses, which will allow us to optimize it in a way that is as efficient and easy to understand as possible.*

*3. Object: Exhibition of Architecture of the Forbidden City.*

*4. The content of the exhibition is the achievements of Lei, an royal architectural designer of the Qing Dynasty, and it displays the architectural relics of the Palace Museum, including drawings, archives, objects and models.*

*5. The exhibition is in the form of online virtual roaming, which requires you to travel in a virtual space. If you have any discomfort with the virtual 3d space experience such as naked eye 3D, VR, panoramic image, virtual roaming, etc., you can give up.*

*6. The data you have filled in will only be used for this study, and you are not required to leave your real name. Personal information will be kept strictly confidential in this study.*

*If you understand the above instructions and agree to participate, you can click on "Exhibition of Architecture of the Forbidden City" to start.*

5. **Authors report a pre-test of the questionnaire and an alpha with 18 participants. Is this useful? What was the reliability index with the full sample?**

Authors’ response: Thank you for your correction. It is our negligence that we did not report the Cronbach’s α coefficient of all valid questionnaires in the study. We have added this on p. 13, lines 5–10.

6. **Data were partial. First, items in the database are in chinese, they should be translated in english to be readable by international audience; second, qualitative data from interviews were missing. These are also mandatory for consideration by PLOS ONE.**

Authors’ response: We apologize for this, and we will translate the database in the future. Qualitative data will also be translated, collated, and uploaded.

7. **Figures and more information on participants' experience and tasks within the exploration would improve the readers' comprehension of the research.**

Authors’ response: Thank you very much for your suggestions. We have added Figure 2 and Figure 3 in p. 12­ to make our tests clearer and easier to understand.

8. **Final discussion says little about the take-home messages for a broader audience: what have we learnt about the design and evaluation of online museums in general?**

Authors’ response: Thank you very much for your reminder. It was an oversight error that we considered more about museum staffs and designers than a broader audience. We have added about this in the conclusion on p. 26, lines 21–23 and p. 27, line 1-5, with implications for a wider audience.

**Journal Requirements**

1. **Please ensure that your manuscript meets PLOS ONE's style requirements, including those for file naming.**

Authors’ response: Thank you very much for your reminder. We have modified our manuscript according to the requirements of the journal to conform to PLOS ONE's style.

2. **PLOS requires an ORCID iD for the corresponding author in Editorial Manager on papers submitted after December 6th, 2016. Please ensure that you have an ORCID iD and that it is validated in Editorial Manager. To do this, go to ‘Update my Information’ (in the upper left-hand corner of the main menu), and click on the Fetch/Validate link next to the ORCID field. This will take you to the ORCID site and allow you to create a new iD or authenticate a pre-existing iD in Editorial Manager.**

Authors’ response: Thank you for your reminding. I have registered the ORCID ID and updated my personal information.

3. **Please include additional information regarding the survey or questionnaire used in the study and ensure that you have provided sufficient details that others could replicate the analyses. For instance, if you developed a questionnaire as part of this study and it is not under a copyright more restrictive than CC-BY, please include a copy, in both the original language and English, as Supporting** **Information.**

Authors’ response: We apologize that it was an oversight error on our part to not upload these questionnaires. We have uploaded the original and translated version of our questionnaire in the Support Information.

4. **Please amend the manuscript submission data (via Edit Submission) to include author Jia Li.**

Authors’ response: Thank you for the reminder. This was an editing error; we have added Jia Li in the edited submission.

5. **Please amend your authorship list in your manuscript file to include author Taiwan Jia.**

Authors’ response: Thank you for the reminder; “Taiwan Jia” was a mistake in editing, and there is no such author. We have modified the personal information again.

6. **Please include captions for your Supporting Information files at the end of your manuscript, and update any in-text citations to match accordingly.**

Authors’ response: Thank you for reminding us. We have added Supporting Information on p. 31 and updated our in-text citations.

**Reviewer #1**

**This is an excellent research paper of relevance to museum studies and it should be published. The methods and process are well-described and clear. The paper is well-written. I am not a statistician so I can't evaluate this part. However, I would be happy to use the paper and evaluation tool in my own teaching in museum studies. My "minor revisions" suggestions are very minor. First, on p. 3, ICOMOS should be defined or the name written out fully. Next, on p. 9, in the chart, at Learning D3 the term "Fun of knowledge" is written. This is not correct English usage. Instead, it should say something like "Enjoyable presentation of information." Then, on p. 22, the term "generality" is used in reference to a suggestion by the authors that the concept should become a "vital evaluation standard." The term's application should be defined and an example included, in my view. Last, on p. 18-19 and again on p. 21 the authors refer to inclusivity, inclusiveness and ability. Yet, they do not mention disability (an oversight, in my view), and the solutions offered through disability studies and inclusive design, such as image descriptions, audio descriptions, and so on. For example, see: https://www.acb.org/adp/ There are many such strategies recommended for museums by disability studies scholars and activists--see Amanda Cachia and Georgina Kleege, for example. The paper should direct museum-based readers to look to these and related sources for suggestions.**

Authors’ response: Thank you very much for your recognition and support, which have encouraged us to continue improving our manuscript. In light of your suggestions, we have made the following revisions.

- We added the full name for the ICOMS on p. 3, line 11.
- We replaced "Fun of information" in D3 in Table 2 (original Table 1) on p. 11 with “Enjoyable presentation of information”.
- “Generality” in p. 27, lines 11–12(original p. 22) was a wrong word, while the correct one should be “inclusivity”. The explanation of inclusivity has been defined on p. 23, line 4-5.
- Thank you very much for your suggestions on inclusivity; we have learnt a lot from the links you provided. We have discussed this more on p. 24, lines 5–20, with references to some relevant literature.

Thank you very much for pointing out the mistakes in out manuscript and putting forward these valuable suggestions, which made our work more reasonable and easier to understand.

**Reviewer #2**

1. **The manuscript discusses a research to develop a heuristic evaluation scale based on the existing literature and a case study. The case study is a virtual online museum tour. The proposed evaluation scale includes four dimensions: authenticity, interaction, navigation, and learning.**

**I am not expert in factor analysis, and I assume that the methodology is properly applied.**

**My concerns are related to the description of the case study and the methods.**

Authors’ response: Thank you very much for your recognition and support.

2. **First of all, I suggest the authors to better describe the case study. In addition to the link provided to access the online tour, there is the need to understand the functionalities, how the experience is structured, the peculiarities of the museum as well as the interaction modalities. Adding screenshots of relevant points of interest can help to better understand the case study.**

Authors’ response: Thank you very much for your suggestions. On p. 7, lines 10–19, we described the functionalities, structure, peculiarities, interaction modalities and other elements of the exhibition. We also illustrated them through Figure 1on p. 7.

3. **Secondly, some details related to the survey are missing:**

**-Since the ethics statement is not provided, I wonder whether the authors deal with the informed consent to involve the participants in the research.**

Authors’ response: Thank you for pointing this out, and we did forget to provide an ethics statement. We have added relevant statements on p. 12, lines 19–21 and p. 13, line 1-3. The participants have fully understood the purpose, process, risk and data use of the study. They filled in the questionnaire anonymously, and the data were recorded with their consent. This study adopted a paperless electronic questionnaire. The questionnaires were filled only with the consent of the participants.

**- How was the EAFC online experience presented to the participants? I wonder whether the authors provided some instructions to the participants about the navigation and the interface, or just let them free to explore the functionalities.**

Authors’ response: Thank you for raising this point, and we have added description on p. 12 of the paper and Figure 2 and Figure 3 to further explain the testing process. Participants were only asked to find the designated artifact at the beginning; the rest of their visit was	undisturbed.

**- What was the task to accomplish during the experience? Was it the same for all the participants?**

Authors’ response: Yes, all the participants had the same task. They all needed to find the artifact named Ceiling of Ci Ning Palace Garden Linxi Pavilion. I apologize for that we forgot to explain in the article. We have added the description and a figure on p. 12, lines 12–16.

**- What were the groups and attributes used for the stratified sampling of the interviewees?**

Authors’ response: Thank you for pointing out this missing part. We did lack description of the groups and attributes used for the stratified sampling of the interviewees. However, we have strengthened the description on p. 16, lines 2–6 and added Table 7 on p. 17.

**- It is not clear the reason why the researcher asked “further random questions” during the interview. Is “random” the proper term?**

Authors’ response: Thank you for your correction. We also think that “random” is not very appropriate. We have re-described the interview questioning process on p. 16, line8-11.

**- Moreover, it is not clear how the qualitative data from the interviews were analysed and integrated with the questionnaire’s results.**

Authors’ response: Thank you for pointing out our oversight error. The questions of our semi-structured interview were formulated based on the scale dimensions of quantitative research, and the discussion was also based on the results of these two parts. We gave explanations on p. 16, line 8–11, and added Table 6 on p. 16 and p.17 for further explanation.

4. **Finally, I suggest the authors to introduce the scale by conceptualizing the different elements, as well as to clearly explain how their scale differs from and improve the existing literature.**

Authors’ response: Thanks for your suggestion; we have conceptualized the different elements in the scale in Table 8 on p. 18–19. In addition, we have listed the scales we refer to in Table 1 on p. 9 to provide the reader with a comparison for the scales we have designed.

5. **A final comment is about the brief discussion about inclusivity, that I appreciate. Indeed it is a relevant issue to make virtual museum accessible for people with diverse needs and abilities. I hope that the authors will further explore this aspect in details, because the literature is missing such investigations to guide the future design.**

Authors’ response: Thank you very much for your recognition. On p. 24, lines 5–20 we have discussed more about the dimension of inclusivity and have cited some relevant literatures, hoping to provide more information to readers.
